# An Improved Codon Modeling Approach for Accurate Estimation of the Mutation Bias

**DOI:** 10.1093/molbev/msac005

**Published:** 2022-01-11

**Authors:** Thibault Latrille, Nicolas Lartillot

**Affiliations:** 1 CNRS, Laboratoire de Biométrie et Biologie Évolutive UMR, Université de Lyon, Université Lyon 1, Villeurbanne, France; 2 École Normale Supérieure de Lyon, Université de Lyon, Université Lyon 1, Lyon, France

**Keywords:** codon models, phylogenetics, nucleotide bias, mutation–selection models

## Abstract

Phylogenetic codon models are routinely used to characterize selective regimes in coding sequences. Their parametric design, however, is still a matter of debate, in particular concerning the question of how to account for differing nucleotide frequencies and substitution rates. This problem relates to the fact that nucleotide composition in protein-coding sequences is the result of the interactions between mutation and selection. In particular, because of the structure of the genetic code, the nucleotide composition differs between the three coding positions, with the third position showing a more extreme composition. Yet, phylogenetic codon models do not correctly capture this phenomenon and instead predict that the nucleotide composition should be the same for all three positions. Alternatively, some models allow for different nucleotide rates at the three positions, an approach conflating the effects of mutation and selection on nucleotide composition. In practice, it results in inaccurate estimation of the strength of selection. Conceptually, the problem comes from the fact that phylogenetic codon models do not correctly capture the fixation bias acting against the mutational pressure at the mutation–selection equilibrium. To address this problem and to more accurately identify mutation rates and selection strength, we present an improved codon modeling approach where the fixation rate is not seen as a scalar, but as a tensor. This approach gives an accurate representation of how mutation and selection oppose each other at equilibrium and yields a reliable estimate of the mutational process, while disentangling the mean fixation probabilities prevailing in different mutational directions.

## Introduction

Phylogenetic codon models are now routinely used in many domains of bioinformatics and molecular evolutionary studies. One of their main applications has been to characterize the genes, sites (Nielsen and Yang 1998; Yang et al. 2005; Murrell et al. 2012), or lineages (Zhang et al. 2005; Kosakovsky Pond et al. 2011) having experienced positive selection (Murrell et al. 2015; Enard et al. 2016). More generally, these models highlight the respective contributions of mutation, selection, genetic drift (Teufel et al. 2018), and biased gene conversion (Kosiol and Anisimova 2019; Pouyet and Gilbert 2021), and the causes of their variation between genes (Zhang and Yang 2015) or across species (Seo et al. 2004; Popadin et al. 2007; Lartillot and Poujol 2011).

Conceptually, codon models take advantage of the fact that synonymous and nonsynonymous substitutions are differentially impacted by selection. Assuming synonymous mutations are neutral, the synonymous substitution rate is equal to the underlying mutation rate (Kimura 1983). Nonsynonymous substitutions, on the other hand, reflect the combined effect of mutation and selection (Ohta 1995). Phenomenological codon models formalize this idea by invoking a parameter *ω*, acting multiplicatively on nonsynonymous substitutions rates (Goldman and Yang 1994; Muse and Gaut 1994). Using a parametric model automatically corrects for the multiplicity issues created by the complex structure of the genetic code and by uneven mutation rates between nucleotides. As a result, *ω* captures the net, or aggregate, effect of selection on nonsynonymous mutations, also called $d_{N}/d_{S}$ (Dos Reis 2015; Spielman and Wilke 2015).

In reality, the selective effects associated with nonsynonymous mutations depends on the context (site-specificity) and the amino acids involved in the transition (Kosiol et al. 2007). Attempts at an explicit modeling of these complex selective landscapes have also been done, leading to mechanistic codon models, based on the mutation–selection formalism (Halpern and Bruno 1998). These models, further developed in multiple inference frameworks (Rodrigue et al. 2010; Tamuri et al. 2012), sometimes using empirically informed fitness landscapes (Bloom 2014), could have many interesting applications, such as inferring the distribution of fitness effects (Tamuri et al. 2012) or detecting genes under adaptation (Rodrigue and Lartillot 2017; Rodrigue et al. 2021), or even phylogenetic inference (Ren et al. 2005). However, they are computationally complex and potentially sensitive to the violation of their assumptions about the fitness landscape (such as site independence). For these reasons, phenomenological codon models remain an attractive, potentially more robust, although still perfectible approach.

The parametric design of phenomenological codon models, relying on a single aggregate parameter *ω* (or site-specific *ω*), raises the question whether they accurately estimate the underlying selective and mutational process. First, simulations under a mutation–selection formalism have shown that the strength of selection is estimated reliably by phenomenological codon models (Spielman and Wilke 2015). More specifically, the model originally proposed by Muse and Gaut (1994), hereafter called MG, gives an accurate estimate of the underlying *ω*. However, several observations suggest that the mutational process is not accurately estimated. For instance, in their simplest form (Goldman and Yang 1994; Muse and Gaut 1994), codon models predict that the nucleotide composition should be the same for all three positions of the codons, and should be equal to the nucleotide equilibrium frequencies implied by the underlying nucleotide substitution rate matrix. In reality, the nucleotide composition differs: the third position shows more extreme GC composition, reflecting the underlying mutation bias, compared with the first and second positions, which are typically closer to 50% GC (Singer and Hickey 2000).

These modulations across the three coding positions have been accommodated using the so-called 3×4 formalism (Goldman and Yang 1994; Pond and Muse 2005), allowing for different nucleotide rate matrices at the three coding positions. However, this is also problematic. For instance, it has the consequence that synonymous substitutions, say from A to C, occur at different rates at the first and third positions. Yet, although modulations of the mutation process along the sequence cannot be excluded, most of the empirically observed compositional differences between positions are likely the consequence of selection, which is stronger at the first and second than at the third position. In principle, these selective effects should not directly impact synonymous rates. Thus, although the mutational process might be more complex, there is no reason to model it in terms of a 3×4 structure which conflates two levels of mechanisms that are not supposed to play together. Simulation experiments suggest that the 3×4 formalism indeed leads to less accurate estimation of *ω* (Spielman and Wilke 2015).

The mutation matrix (1×4) or matrices (3×4) estimated by codon models are thus not correctly reflecting the mutation rates between nucleotides (Rodrigue et al. 2008; Kosakovsky Pond et al. 2010). Instead, what these matrices are capturing is the result of the compromise between mutation and selection at the level of the realized nucleotide frequencies. Conceptually, it is a clear symptom that mutation rates and fixation probabilities are not correctly teased apart by current codon models.

Practically, this misconception could have important consequences in the current interest in investigating the variation between species in GC content, and its effect on the evolution of protein-coding sequences. An important factor here is biased gene conversion toward GC (called gBGC), which can confound the tests for detecting positive selection and, more generally, the estimation of *ω* (Galtier et al. 2009; Ratnakumar et al. 2010; Lartillot et al. 2013; Figuet et al. 2014; Bolívar et al. 2019). Even in the absence of gBGC, however, uneven mutation rates varying across species can have an important impact on the estimation of the strength of selection (Guéguen and Duret 2018). All this suggests that, even before introducing gBGC in codon models, correctly formalizing the interplay between mutation and selection in current codon models would be an important first step, which is the focus of this manuscript.

In this direction, the key point that needs to be correctly formalized is the following. If the nucleotide’s realized frequencies are the result of a compromise between mutation and selection, then this implies that the strength of selection is not the same between all nucleotide or amino acid pairs. For instance, if the mutation process is AT-biased, then, because of selection, the realized nucleotide frequencies at equilibrium will be less AT-biased than expected under the pure mutation process. However, this implies that, at equilibrium, there will be a net mutation pressure toward AT, which has to be compensated for by a net selection differential toward GC.

In order for a codon model to correctly formalize this subtle interplay between mutation and selection, the parameter responsible for absorbing the net effect of selection (i.e., *ω*) should not be a scalar, but an array of *ω* values (i.e., a tensor) unfolding along multiple directions. In the present work, we address the question of whether we can derive a model which is able to correctly tease apart mutation rates and selection without having to explicitly model the underlying fitness landscape. In order to derive a codon model along those lines, our strategy is to first assume a true site-specific evolutionary process, following the mutation–selection formalism. Then, we derive the mean substitution process implied across all sites by this mechanistic model and identify the mean fixation probabilities appearing in this mean-field (MF) process with the array of *ω* tensor to be estimated. Based on this approach, we show that the simplest model that correctly teases apart mutation and selection requires a different value of *ω* for each distinct pair of amino acids. Similar multirate models have been introduced previously (Delport et al. 2010), although never in connection with the question of how to separately infer mutation rates and the mean effect of selection.

## Results

To illustrate the problem, we first conduct simulation experiments under a simple mutation–selection substitution model assuming site-specific amino acid preferences. We use these simulation experiments to explore through summary statistics the intricate interplay between mutation and selection. Then, we explore how codon models with different parameterizations are able to infer the mutation rates and the strength of selection on these simulated alignments. Finally, these alternative models are applied to empirical data.

### Simulation Experiments

Simulations of protein-coding DNA sequences were conducted under an origination–fixation substitution process (McCandlish and Stoltzfus 2014) at the level of codons (see Simulation Model). We assume a simple mutation process with a global parameter controlling the mutational bias toward AT, denoted $\lambda =(\sigma_{A}+\sigma_{T})/(\sigma_{C}+\sigma_{G})$, where *σ_x_* is the equilibrium frequency of nucleotide *x*. This mutational process is shared by all sites of the sequence. With regards to selection, synonymous mutations are considered neutral, such that the synonymous substitution rate equal to the underlying mutation rate. At the protein level, selection is modeled by introducing site-specific amino acid fitness profiles (i.e., a vector of 20 fitnesses for each coding site), which are scaled by a relative effective population size $N_{r}$. A high $N_{r}$ induces site-specific profiles having a large variance, with some amino acids with a high-scaled fitness, whereas all other have a low-scaled fitness. Conversely, a low value for $N_{r}$ induces more even amino acid fitness profiles (i.e., neutral) at each site. Thus, ultimately, the stringency of selection increases with $N_{r}$. Altogether, the two parameters of the model tune the mutation bias (*λ*) and the stringency of selection ($N_{r}$), respectively. All simulations presented in the main manuscript are obtained using the same underlying tree topology and branch lengths of 61 primates from Perelman et al. (2011), along with the same 4,980 codon sites with amino acid fitness profiles resampled from experimentally determined profiles in Bloom (2017).

**Fig. 1.**
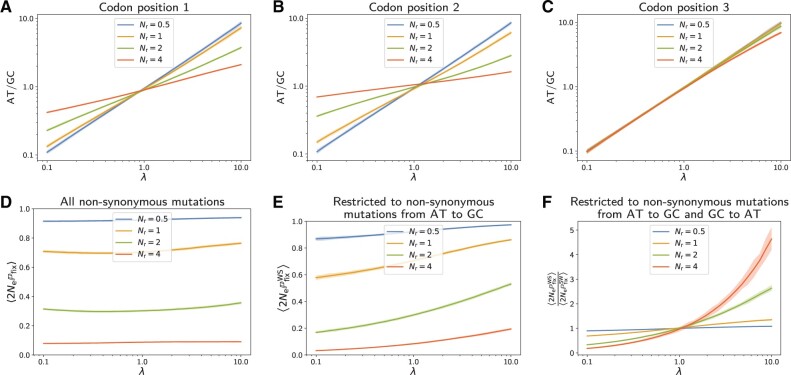
Simulations of 61 primates taxa, 4,980 codon sites, with 100 replicates. Solid lines represent the mean value over the replicates, and the colored area the 95% interquantile range. Top row (*A–C*): observed AT/GC composition of simulated alignment (first, second, and third coding positions), as a function the underlying mutational bias toward AT (*λ*), under different stringencies of selection (different values of relative effective population size $N_{r}$). Bottom row (*D*, *E*): mean-scaled fixation probability of nonsynonymous mutations along simulations, $\langle 2N_{e}P_{\text{fix}}\rangle$, for all mutations (*D*) and for AT-to-GC mutations only (*E*), as a function of the mutational bias (*λ*), under different relative effective population sizes ($N_{r}$). (*F*) Ratio of mean-scaled fixation probability for AT-to-GC over GC-to-AT mutations, as a function of the mutational bias and under different stringencies of selection ($N_{r}$). Mutational bias is balanced by selection in the opposite direction, where this effect increases with the stringency of selection.

Simulation under this origination–fixation process along a species tree results in a multiple sequence alignment of coding sequences for the extant species, from which summary statistics can then be computed. One such straightforward summary statistic is the frequency of the different nucleotides, and the resulting nucleotide bias AT/GC observed in the alignment. This observed nucleotide bias can be computed separately for each coding position (first, second, and third) and compared with the true underlying mutational bias *λ*. As can be seen from figure 1, the third position of codons (fig. 1*C*) reflects the underlying mutational bias quite faithfully, whereas the first and second positions (fig. 1*A* and *B*) are impacted by the strength of selection and display nucleotide biases that are less extreme than the one implied by the mutational process. This differential effect across the three coding positions is explained by nucleotide mutations at the third codon position being more often synonymous, whereas mutations at the first and second positions are more often changing the amino acid and are thus more often under purifying selection.

Apart from the nucleotide bias observed in the alignment, a statistic directly relevant for measuring the intrinsic effect of selection is the mean-scaled fixation probability of nonsynonymous mutations, called $\langle 2N_{e}P_{\text{fix}}\rangle$. This summary statistic $\langle 2N_{e}P_{\text{fix}}\rangle$ can be quantified from the substitutions recorded along the simulation trajectory (see Mean-Scaled Fixation Probability). For very long trajectories, it identifies with the ratio of nonsynonymous over synonymous substitution rates ($d_{N}/d_{S}$ or *ω*) induced by the underlying mutation–selection model (Dos Reis 2015; Spielman and Wilke 2015; Jones et al. 2017). As expected, $\langle 2N_{e}P_{\text{fix}}\rangle$ is always lower than 1 for simulations at equilibrium, under a time-independent fitness landscape (Spielman and Wilke 2015). Quite expectedly $\langle 2N_{e}P_{\text{fix}}\rangle$ decreases with the $N_{r}$ (fig. 1*D*). On the other hand, $\langle 2N_{e}P_{\text{fix}}\rangle$ depends weakly on the mutational bias (*λ*).

The proxy of selection represented by $\langle 2N_{e}P_{\text{fix}}\rangle$ concerns all nonsynonymous mutations, but we can also consider the mean-scaled fixation probability only for the subset of nonsynonymous mutations from weak nucleotides (A or T) to strong nucleotides (G or C), called $\langle 2N_{e}P_{\text{fix}}^{\text{WS}}\rangle$. Interestingly, $\langle 2N_{e}P_{\text{fix}}^{\text{WS}}\rangle$ increases with the strength of the mutational bias toward AT (fig. 1*E*). This distortion of the selective effects toward GC is stronger under an increased stringency of selection, under a higher $N_{r}$. Likewise, the nonsynonymous mutations could also be restricted from strong (GC) to weak nucleotides (AT). This ratio decreases with the strength of the mutational bias toward AT (not shown). As a result, the ratio between $\langle 2N_{e}P_{\text{fix}}^{\text{WS}}\rangle$ and $\langle 2N_{e}P_{\text{fix}}^{\text{WS}}\rangle$ is higher than 1 under a mutational bias toward AT (and lower than 1 respectively for a bias toward GC). It is monotonously increasing with the mutational bias toward AT (fig. 1*F*). Altogether, fixation probabilities are opposed to mutational bias, and the realized equilibrium frequencies are thus at an equilibrium point between these two opposing forces.

### Parameter Inference on Simulated Data

From an alignment of protein-coding DNA sequences, without knowing the specific history of substitutions, can one estimate the mutational bias (*λ*) and the mean-scaled fixation probability $\langle 2N_{e}P_{\text{fix}}\rangle$? In other words, can we tease apart mutation and selection?

**Fig. 2.**
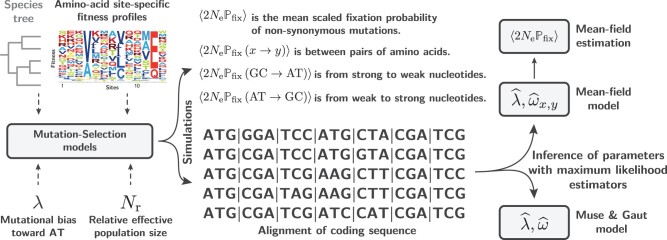
Overall procedure for simulation under a site-specific mutation–selection codon model and inference using a homogeneous codon models. The value of the mutational bias (*λ*) used for simulations can be compared with the value estimated by the codon models ($\hat{\lambda }$) once fitted to the simulated alignment. The mean-scaled fixation probability of nonsynonymous mutations ($\langle 2N_{e}P_{\text{fix}}\rangle$) is recorded along the simulation trajectory, and is directly comparable with $\hat{\omega }$ such as estimated by codon models.

To address this question, here we consider two codon models for inference, differing only by their parametrization of the codon matrix ***Q***, which we first test against simulated data (see fig. 2). Both are homogeneous along the sequence (i.e., not site-specific). The first is based on Muse and Gaut (1994) formalism and uses a scalar *ω* parameter, whereas the second is based on a tensor representation of *ω*.

#### ω As a Scalar: The Muse and Gaut Formalism

This model is defined in terms of a generalized time-reversible nucleotide rate matrix ***R*** and a scalar parameter *ω*. The matrix ***R*** is a function of the nucleotide frequencies σ and the symmetric exchangeability rates ρ (Tavaré 1986):

(1) $$R_{a,b}=\rho_{a,b}\sigma_{b}.$$

At the level of codons, the substitution rate between the source (*i*) and target codons (*j*) depends on the underlying nucleotide change between the codons M(i,j) (e.g., M(AAT,AAG)=TG), and whether or not the change is nonsynonymous. Altogether, the substitution rates between codons $Q_{i,j}$, formalized by Muse and Gaut (1994) are defined as follows:

(2) $$\{\begin{array}{cc} Q_{i,j} & =0\;\text{if codons}\;i\;\text{and}\;j\;\text{are more than one mutation away},\\ Q_{i,j} & =R_{M(i,j)}\;\text{if codons}\;i\;\text{and}\;j\;\text{are synonymous},\\ Q_{i,j} & =\omega R_{M(i,j)}\;\text{if codons}\;i\;\text{and}\;j\;\text{are nonsynonymous}. \end{array}$$

The model can be fitted by maximum likelihood. Then, from the estimate of $\hat{R}$, one can derive a nucleotide bias toward AT as:

(3) $${\hat{\lambda }}_{\text{MG}}=(\hat{\sigma_{A}}+\hat{\sigma_{T}})/(\hat{\sigma_{G}}+\hat{\sigma_{C}}).$$

As for the mean strength of selection $\langle 2N_{e}P_{\text{fix}}\rangle$, a direct estimate is given by $\hat{\omega }$.

**Fig. 3.**
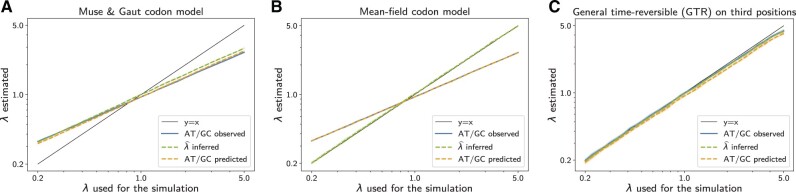
Simulations with 61 primates taxa and 4,980 codon sites. Estimated versus true mutational bias, using a codon model in which *ω* is modeled as a scalar (MG formalism, MG, panel *A*) or as a tensor (MF approach, panel *B*), or by applying a GTR nucleotide model to the 4-fold degenerate third-coding positions only (panel *C*).

As shown in figure 3*A*, estimate of the mutational bias is halfway between the nucleotide bias observed in the alignment and the true mutational bias used during the simulation. Thus, the MG model cannot reliably infer the mutational bias. On the other hand, $\hat{\omega }$ is close to the underlying mean-scaled fixation probability $\langle 2N_{e}P_{\text{fix}}\rangle$ computed during the simulation (61 primates taxa, 4,980 codon sites, 100 replicates), with a precision of 97.2%. Thus, the failure to correctly estimate the mutation process does not seem to have a strong impact on the estimation of the overall strength of selection, at least in the present case.

#### ω As a Tensor: MF Derivation

We would like to derive a codon model that would be more accurate than the MG model concerning the estimation of the mutation bias, but that would still be site-homogeneous. However, the true process is site-specific. The link between the two can be formalized by projecting the site-specific processes onto a gene-wise process, using what can be seen as a MF approximation (Goldstein and Pollock 2016). The gene-wise process obtained by this procedure is expressed in terms of mutation rates and mean-scaled fixation probabilities. Finally, the mean-scaled fixation probabilities can be identified with the *ω*-tensor.

Specifically, at each site z, the underlying codon process is:

(4) $$\{\begin{array}{cc} Q_{i,j}^{\left(z\right)} & =0\;\text{if codons}\;i\;\text{and}\;j\;\text{are more than one mutation away},\\ Q_{i,j}^{\left(z\right)} & =R_{M(i,j)}\;\text{if codons}\;i\;\text{and}\;j\;\text{are synonymous},\\ Q_{i,j}^{\left(z\right)} & =R_{M(i,j)}2N_{e}P_{\text{fix}}^{\left(z\right)}(i,j)\;\text{if codons}\;i\;\text{and}\;j\;\text{are nonsynonymous}. \end{array}$$

Where $2N_{e}P_{\text{fix}}^{\left(z\right)}(i,j)$ is the scaled fixation probability of codon *j* against codon *i*, at site z. At equilibrium of the process, averaging over sites under the equilibrium distribution gives the MF gene-level process:

(5) $$\{\begin{array}{cc} \langle Q_{i,j}\rangle & =0\;\text{if codons}\;i\;\text{and}\;j\;\text{are more than one mutation away},\\ \langle Q_{i,j}\rangle & =R_{M(i,j)}\;\text{if codons}\;i\;\text{and}\;j\;\text{are synonymous},\\ \langle Q_{i,j}\rangle & =R_{M(i,j)}\langle 2N_{e}P_{\text{fix}}(i,j)\rangle \;\text{if codons}\;i\;\text{and}\;j\;\text{are nonsynonymous}. \end{array}$$

However, because selection between codons reduces to selection between pairs of amino acids, $\langle 2N_{e}P_{\text{fix}}(i,j)\rangle$ only depends on the amino acids encoded by *i* and *j* (see Derivation of MF Model in Materials and Methods). Thus, by identification, the inference model should be parameterized by a set of *ω* values for all pairs of amino acids, denoted $\omega_{x,y}$. For 20 amino acids, the total number of pairs of amino acids is 190, hence 380 parameters by counting in both directions. However, because of the structure of the genetic code, there are 75 pairs that are one nucleotide away, since some amino acids are not directly accessible through a single nonsynonymous mutation. As a result, the number of parameters necessary to determine all nonzero entries of the tensor ($\omega_{x,y}$) in both directions is 150. Finally, under the assumption of a reversible process, the number of parameters can be reduced to 75 symmetric exchangeabilities ($\beta_{x,y}$) and 20 stationary effects (*ϵ_x_*):

(6) $$\omega_{x,y}=\epsilon_{y}\beta_{x,y},\;\text{where}\;\beta_{x,y}=\beta_{y,x}.$$

Altogether, the substitution rates between codons $Q_{i,j}$ are defined as:

(7) $$\{\begin{array}{cc} Q_{i,j} & =0\;\text{if codons}\;i\;\text{and}\;j\;\text{are nonneighbors},\\ Q_{i,j} & =R_{M(i,j)}\;\text{if codons}\;i\;\text{and}\;j\;\text{are synonymous},\\ Q_{i,j} & =R_{M(i,j)}\omega_{A(i),A(j)}\;\text{if codons}\;i\;\text{and}\;j\;\text{are nonsynonymous}, \end{array}$$

where A(i) is the amino acid encoded by codon *i* and $\omega_{x,y}$ is given by equation (6).

This MF model is fitted by maximum likelihood, giving an estimate for its parameters, $\hat{R},\;\hat{\beta }$ and $\hat{\epsilon }$. Then, from the estimate of the GTR nucleotide matrix ($\hat{R}$), a mutation bias ${\hat{\lambda }}_{\text{MF}}$ can be estimated as previously (eq. 3 above).

As shown in figure 3*B*, and under a variety of scenarios (number of sites, branch lengths, tree topology) in Supplementary Material online, ${\hat{\lambda }}_{\text{MF}}$ under the MF model provides an accurate estimate of the true mutational bias. In other words, the MF model can tease out the observed AT/GC bias of the alignment and the underlying mutational bias Interestingly, in spite of invoking a single mutation bias across all nucleotide sites, the MF model predicts distinct nucleotide frequencies at the three coding positions (Supplementary Material online). These predicted frequencies match the frequencies that are observed on the alignment. In other words, the MF model is able to explain how a site-homogeneous mutational process combined with a selective pressure acting at the amino acid level can in the end produce a 3×4 pattern of nucleotide frequencies.

**Fig. 4.**
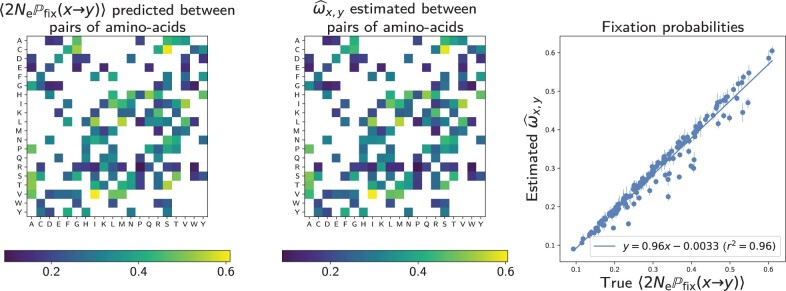
True versus estimated values of $\omega_{x,y}$ between pairs of amino acids under our MF model. The true values are given by equation (26). Simulations on 61 primates taxa with 4,980 codon sites over 100 replicates. Vertical bars are the 95% confidence intervals for the mean value.

The mean-scaled fixation probability of nonsynonymous mutations $\langle 2N_{e}P_{\text{fix}}\rangle$ can also be computed. It is now a compound parameter, expressed as a function of $\hat{R},\;\hat{\beta }$ and $\hat{\epsilon }$ (see Mean-Scaled Fixation Probability $\langle 2N_{e}P_{\text{fix}}\rangle$ under the MF Model). Under this model, $\langle 2N_{e}P_{\text{fix}}\rangle$ is close to the true mean-scaled fixation probability $\langle 2N_{e}P_{\text{fix}}\rangle$ computed during the simulation, with a precision of 96.9% (61 primates taxa, 4,980 codon sites, 100 replicates). Moreover, as shown in figure 4, the estimated rates ${\hat{\omega }}_{x,y}$ between pairs of amino acids is congruent with the predicted mean-scaled fixation probability computed analytically as a function of the underlying site-specific fitness profiles and the mutation matrix as in equation (26).

More analyses are shown in Supplementary Material online, with different sequence length (498, 996, 2,490, 4,980 and 9,960 codon sites), different branch lengths (decreased by a factor 2 and increased by a factor 2, 4, 8), and a different topology (90 mammals). These analyses suggest that the number of sites does not influence the estimator’s accuracy for mutational bias ($\hat{\lambda }$), nor for selection pressure ($\hat{\omega }$). Finally, for large sequence divergence (Supplementary Material online), saturation of sequences (multiple substitutions at the same site) leads to less accurate estimation: both the MG and MF models fail to give an accurate estimator of $\hat{\omega }$. The mutation bias $\hat{\lambda }$, on the other hand, is still correctly estimated under the MF model.

### Estimation on Empirical Sequence Data

The two alternative models of inference just considered, namely the Muse and Gaut (MG) and the MF codon models, were then applied to empirical protein-coding sequence alignments. Several examples were analyzed: the nucleoprotein in *Influenza Virus* (as human host) assembled in Bloom (2017), the *β*-lactamase in *bacteria* gathered in Bloom (2014), as well as orthologous AT-rich genes (such as to prevent the confounding effect of gBGC) in primates extracted from the OrthoMam database (Scornavacca et al. 2019) as shown in table 1.

**Table 1.** Mutational Bias (*λ*) and Mean-Scaled Fixation Probability ($\langle 2N_{e}P_{\text{fix}}\rangle$) Estimated under the MG and MF Models on Distinct Concatenated DNA Alignments of Orthologous Genes.

	*β*-Lactamase	Nucleoprotein	Primates AT-Rich
Data Set	Bloom	Bloom	Scornavacca et al.
Number of taxa	85	180	22
Number of sites	263	498	4,877
AT/GC	0.792	1.154	2.028
AT/GC at first position	0.583	1.057	1.303
AT/GC at second position	1.177	1.221	2.541
AT/GC at third position	0.714	1.192	2.648
MG mutational bias (${\hat{\lambda }}_{\text{MG}}$)	0.853	1.447	2.073
MF mutational bias (${\hat{\lambda }}_{\text{MF}}$)	0.690	1.748	2.419
MG $\hat{\omega }$	0.332	0.114	0.526
MF $\langle 2N_{e}P_{\text{fix}}\rangle$	0.336	0.116	0.525
MF $\langle 2N_{e}P_{\text{fix}}^{\text{WS}}\rangle$	0.297	0.141	0.594
MF $\langle 2N_{e}P_{\text{fix}}^{\text{SW}}\rangle$	0.412	0.092	0.487
Δ AIC	37.6	165.2	1,527.0
$P\left(\chi_{df=\mathrm{93}}^{2}>LRT\right)$	9.2$\times 10^{-13}$	1.2$\times 10^{-31}$	3.9$\times 10^{-296}$

Note.—The MF model contains 95 parameters with 75 amino acid exchangeabilities and 20 amino acid equilibrium frequencies. However, we have two constrains that reduce the degree of freedom; the sum of all 20 amino acid equilibrium frequencies equals 1 and the sum of all 61 codon frequencies equals 1.

For alignments globally biased toward AT (nucleoprotein and AT-rich concatenate in primates), similarly to what was observed in the simulation experiments presented above, the mutational bias estimates under the two codon models are greater than the observed nucleotide bias (i.e., $1<\text{AT}/\text{GC}<\hat{\lambda }$). This effect is, as previously, probably due to selection at the level of amino acids, partially opposing the mutational bias. More importantly, the mutational bias estimated by the MF model is more extreme than the MG estimate (i.e., $1<{\hat{\lambda }}_{\text{MG}}<{\hat{\lambda }}_{\text{MF}}$). These examples behave identically to the observations made with simulated alignments, where, compared with MG, the MF model estimates a stronger mutational bias, which was also closer to the real value. Thus, a reasonable interpretation is that MG is also underestimating the underlying mutational bias in the present case, and that the estimate of the MF model is more accurate.

Concerning selection, the estimated mean-scaled fixation probability of nonsynonymous mutations, is similarly estimated in the MF and MG models ($\langle 2N_{e}P_{\text{fix}}\rangle ≃\hat{\omega }$). Additionally, in the MF model, $\langle 2N_{e}P_{\text{fix}}\rangle$ can be restricted to mutations from weak nucleotides (AT) to strong (GC), or vice versa (see Mean-Scaled Fixation Probability $\langle 2N_{e}P_{\text{fix}}\rangle$ under the MF Model). We observe that under a mutational bias favoring AT (i.e., λ>1), the mean fixation probability of nonsynonymous mutations is higher toward GC than toward AT, $\langle 2N_{e}P_{\text{fix}}^{\text{WS}}\rangle >\langle 2N_{e}P_{\text{fix}}^{\text{SW}}\rangle$, as expected under a AT-biased mutation process.

Reciprocally, for alignment globally biased toward GC (*β*-lactamase), the estimated mutation bias is stronger (toward GC) than the bias observed on the alignment (i.e., ${\hat{\lambda }}_{\text{MF}}<\text{AT}/\text{GC}<1$). Curiously, in *β*-lactamase, the MG model estimates a weaker underlying mutational bias than the observed bias (i.e., $\text{AT}/\text{GC}<{\hat{\lambda }}_{\text{MG}}<1$). This effect could be due to the first, second, and third positions having compositional biases in different directions, which is harder to disentangle (table 1). Concerning selection, we observe that the fixation probability of nonsynonymous mutations is higher on average toward AT than toward GC, $\langle 2N_{e}P_{\text{fix}}^{\text{SW}}\rangle >\langle 2N_{e}P_{\text{fix}}^{\text{WS}}\rangle$, as expected under a GC-biased mutation process.

The results obtained on empirical data are globally in agreement with the observations gathered from the simulation experiments, namely that the presence of a mutational bias results in a selection differential, taking the form of a slightly higher mean fixation probability of nonsynonymous mutations opposing the mutational bias. Moreover, by setting ϵ=1 and β=ω×1 in our MF model, we retrieve the nested MG model, hence, both models are directly comparable.

The empirical fit to the data between the nested models, using AIC and Likelihood ratio tests (Posada and Buckley 2004) favor the MF model compared with the MG model (table 1). Of note, owing to its unreasonable assumption that $\langle 2N_{e}P_{\text{fix}}\rangle$ is the same across all amino acid pairs, the MG model is in fact very easy to improve upon (Delport et al. 2010), and thus the higher fit of MF compared with MG is not in itself a very strong argument in favor of the use of MF. However, our simulations suggest that, in spite of the larger estimation error on the individual rates between all pairs of amino acids on smaller alignments, the estimate of the mutation bias is always reasonably accurate, even on small alignments (Supplementary Material online).

In another simulation analysis, it has also been shown that better fitting models could sometimes lead to less accurate inference (Spielman and Wilke 2015). This point was more specifically made concerning models such as 3×4. We concur with this argument, which is particularly relevant in the present context. The 3×4 model is typically better fitting than the 1×4 model (which is the default considered here through the MG model). Yet, and this is precisely one of the main points of the present work, 3×4 does not represent the correct way to model the processes that are creating the variation in nucleotide frequencies across the three coding positions and, for that reason should not be used, in spite of its higher fit. The MF model, on the other hand, gives the correct logical solution to this problem and our simulation experiments confirm that this leads to accurate estimation of the mutation bias. In summary, this is the conjunction of the higher fit observed here on empirical data with the logical arguments and the simulation experiments presented above that together justify the use of the MF model. Based on these justifications, we can thus interpret the estimate of ${\hat{\lambda }}_{\text{MF}}$ as reflecting the mutation bias, and the difference between $\langle 2N_{e}P_{\text{fix}}^{\text{SW}}\rangle$ and $\langle 2N_{e}P_{\text{fix}}^{\text{WS}}\rangle$ as suggesting that the fixation biases are different in the two directions also in the case of empirical data.

Altogether, our MF model is favored by empirical data sets, and simultaneously estimates more extreme (and probably more accurate) mutational biases compared with the MG model.

## Discussion

In protein-coding DNA sequences, the nucleotide composition results from a subtle interplay between mutation at the level of nucleotides and selection at the protein level. As a result, the nucleotide bias observed in the alignment is different from the underlying mutational bias. However, current parametric codon models predict that the observed and underlying mutational biases should be equal. For that reason, they are inherently misspecified and are unable to tease apart opposing effects of mutation and selection correctly. As shown in our work, the misspecification of these models does not strongly impact the estimation of the net effect of selection on nonsynonymous mutations ($\hat{\omega }$). This novel result is important, as it is reassuring for a certain number of previously published analyses, in particular correlating $\hat{\omega }$ with life-history traits, in a context where GC content also correlates with life-history traits (Figuet et al. 2016; Bolívar et al. 2019). However, current parametric models do not estimate the mutational process accurately.

In this work, we sought to find the simplest parametric codon model able to correctly tease apart mutation rates on one hand, and net mean fixation probabilities on the other hand, and this, without having to explicitly model the underlying fitness landscape. In order to derive a codon model along those lines, our strategy is to first assume an underlying microscopic model of sequence evolution (here, a mutation–selection model based on a site-specific, time-independent fitness landscape). Then, we derive the gene-wise mean fixation probabilities between all pairs of codons, implied by the underlying microscopic process. Finally, we observe that this MF process should in fact invoke as many distinct *ω* parameters as there are pairs of amino acids that are nearest neighbors in the genetic code. There are reversibility conditions, reducing the dimensionality and allowing for a GTR-like parameterization of this tensor (95 parameters for selection).

Inferring parameters on simulated alignments, we show that the model derived using this MF argument correctly estimates the underlying mutational bias and selective pressure. In this respect, our work gives the first clear explanation of how to correctly disentangle the underlying mutational bias and the observed nucleotide frequencies. Our model can predict the accurate nucleotide composition at first, second, and third codon positions, whereas current parametric models fail to predict them. We argue that parametric codon models using three different mutational processes at the first, second, and third coding positions (3×4 formalism) to accommodate for variation in observed nucleotide frequencies is not a theoretically sound modeling. Indeed this variation is an emerging property of the balance between mutation and selection as shown in our work. The 3×4 formalism has previously been shown to lead to inaccurate inference of $\hat{\omega }$ (Spielman and Wilke 2015). Altogether, we concur in this direction that 3×4 formalism is inaccurate and not mechanistically sound, and as a result should not be used to estimate $\hat{\omega }$.

Applying our model to empirical alignments, we also observe that there is a selection differential opposing the mutational bias. This observation also points to a fundamental property of natural genetic sequences, namely that they are not optimized but are the result of interactions between evolutionary forces (Sella and Hirsh 2005). In the specific case highlighted in this work, the mutational bias at the nucleotide-level results in suboptimal amino acids being overrepresented in the sequence, compared with what would be expected based on their fitness alone. For example, under a mutational bias toward AT, AT-rich amino acids might not necessarily be the fittest but are excessively generated by the mutational process, resulting in a stronger purifying selection against AT-rich amino acids. This was pointed out previously (Singer and Hickey 2000), although never directly formalized in a phylogenetic codon model. One important consequence of this tradeoff between mutation and selection is that the observed higher mean fixation probability toward GC is mimicking the effect of biased gene conversion toward GC (gBGC), although unlike gBGC, the phenomenon described here corresponds to a genuine selective effect. Although we did not explore the consequences of this at the level of intraspecific polymorphism, the selection differential uncovered here also implies that the distribution of fitness effects is not the same in the two directions, either toward AT or toward GC. Specifically, in the presence of an AT-biased mutation process, the nonsynonymous GC polymorphisms are expected to segregate at higher frequencies, compared with nonsynonymous AT polymorphisms.

These observations have some practical implications: for instance, experiments observing a fixation (or segregation) bias toward GC at the nonsynonymous level must also rule out that this fixation bias is not a simple consequence of the tradeoff between mutation and selection. More generally, our observations and modeling principles offer a useful preliminary basis to better understand how mutation and selection will work together with GC-biased gene conversion (gBGC), and therefore will help better understand how gBGC will impact both nucleotide composition and $\hat{\omega }$. It is worth mentioning that in our result, we focused on the fixation probability from AT to GC, $\langle 2N_{e}P_{\text{fix}}^{\text{WS}}\rangle$, because of the relationship to gBGC. However, in practice, the same analysis and methods can be applied to any subset of nucleotides or codons.

Our MF parametric model uses gene-level parameters (in the form of a tensor) that is meant to capture the mean-scaled fixation probabilities. This derivation, and its validation on simulated data, shows that, even though the underlying selective landscape is site-specific, a gene-level approximation can nonetheless accurately disentangle mutation and selection. As a result, this study demonstrates that phenomenological models derived out of mechanistic models are more compact (i.e., not site-specific), and in certain cases are sufficient to extract the relevant parameters.

The methodology proposed here for deriving inference models consists in proceeding in two steps, first assuming an underlying mechanistic model of sequence evolution, parameterized by variables that are derived from first principles (fitness landscape, mutation rates, …). Subsequently, the phenomenological inference model is obtained by matching its parameters (here, the entries of the *ω* tensor) with the aggregate parameters derived from the application of the MF procedure to the mechanistic model. Altogether, we believe that the approach used here could be applied more generally: inference models can be phenomenological in practice, but should nonetheless be derived from an underlying mechanistic model, so as to correctly formalize the interplay between mutation, selection, drift, and other evolutionary forces.

Our phylogenetic codon model is not the first to model *ω* as a tensor. Thus, Yang et al. (1998) introduced a codon model in which *ω* depends on the distance between amino acids, measured in terms of the Grantham (1974) distance. Additionally, Tang and Wu (2006) leveraged *ω* tensors in order to detect positively selected genes. The novelty of the present work is to formalize the articulation between the nucleotide composition, the mutational bias, and selection between different amino acids. Finally, this work is still preliminary since the MF model should be tested against a more diverse range of empirical data, in terms of phylogenetic depth, strength of selection, and codon usage bias to assert the validity of our empirical results. In addition, several other parametrization of codon models as listed in Rodrigue et al. (2008) and Kosakovsky Pond et al. (2020) should be included in a broader comparison of the accuracy of the estimation of the underlying mutational bias and strength of selection on protein-coding DNA sequences.

## Materials and Methods

### Simulation Model

We seek to simulate the evolution of protein-coding sequences along a specie tree. Starting with one sequence at the root of the tree, the sequences evolve independently along the different branches of the tree by point substitutions, until they reach the leaves. At the end of the simulation, we get one sequence for each leaf of the tree, meaning one sequence per species. The substitution is modeled using the origination–fixation approximation, that is, substitution rates are the product of the mutation rate at the nucleotide level, and fixation probabilities, based on selection at the amino acid level.

The mutation process is assumed homogeneous across sites. On the other hand, selection is assumed to be varying along the sequence. During the simulation, given the current sequence, the substitution rates toward all possible mutants (one nucleotide change) are computed and the next substitution event is drawn randomly based on Gillespie’s algorithm (Gillespie 1977).

### Mutational Bias at the Nucleotide Level

The mutation rate between nucleotides is always proportional to *μ*. Moreover, mutations from any nucleotide to another weak nucleotide are increased by the factor *λ* compared with mutations to another strong nucleotide. The mutation rate matrix is thus:

(8) $$R=\begin{array}{c} \begin{array}{ccccc} A & C & & G & T \end{array}\\ \begin{array}{c} A\\ C\\ G\\ T \end{array}\left(\begin{array}{cccc} -\mu (2+\lambda ) & \mu & \mu & \mu \lambda \\ \mu \lambda & -\mu (1+2\lambda ) & \mu & \mu \lambda \\ \mu \lambda & \mu & -\mu (1+2\lambda ) & \mu \lambda \\ \mu \lambda & \mu & \mu & -\mu (2+\lambda ) \end{array}\right) \end{array}$$

Which has the following stationary distribution:

(9) σR=1,

(10) $$\Leftrightarrow \sigma =\left(\frac{\lambda }{2+2\lambda },\frac{1}{2+2\lambda },\frac{1}{2+2\lambda },\frac{\lambda }{2+2\lambda }\right).$$

As a result, the ratio of weak over strong nucleotide frequencies at stationarity is equal to *λ*:

(11) $$\frac{\sigma_{A}+\sigma_{T}}{\sigma_{C}+\sigma_{G}}=\frac{\lambda {\left(2+2\lambda \right)}^{-1}+\lambda {\left(2+2\lambda \right)}^{-1}}{{\left(2+2\lambda \right)}^{-1}+{\left(2+2\lambda \right)}^{-1}},\;\text{from}\;\text{equation}\;(10),$$

(12) =λ.

*μ* is constrained such the expected flow ($-\sum_{a}\sigma_{a}R_{a,a}$) of mutation equals to 1.

### Selection at the Amino Acid Level

The substitution rate is considered null between any two codons differing by more than one nucleotide. Otherwise, the mutation rate between a pair of codons is given by the mutation rate of the underlying single nucleotide change. Selection is modeled at the amino acid level, that is, we assume that all codons encoding for one particular amino acid are selectively equivalent.

To take into account the heterogeneity of selection between different sites of the protein, we assume that each site z of the sequence is independently evolving under a site-specific fitness landscape, characterized by a 20D frequency vector of scaled (Wrightian) fitness parameters $\psi^{\left(z\right)}=\{\psi_{a}^{\left(z\right)},\;1\leq a\leq 20\}$. The fitness vectors $\psi^{\left(z\right)}$ used in this study are extracted from Bloom (2017), which were experimentally determined by deep mutational scanning for 498 codon sites of the nucleoprotein in *Influenza Virus* strains (as human host). For each codon site z of our simulation, we assign randomly one the 498 fitness profile (sampling with replacement) experimentally determined, which altogether determines the (Wrigthian) fitness vectors across sites. The malthusian fitness (or log-fitness) of amino acid a, denoted $F_{a}^{\left(z\right)}$, is scaled by the relative effective population size ($N_{r}$) accordingly:

(13) $$F_{a}^{\left(z\right)}=N_{r}\ln\left(\psi_{a}^{\left(z\right)}\right),\;z\in \{1,\ldots ,Z\},\;a\in \{1,\ldots ,20\}$$

At site z, the substitution rate between nonsynonymous codons *i* and *j* is given by the product of the mutation rate and the probability of fixation:

(14) $$Q_{i,j}^{\left(z\right)}=R_{M(i,j)}\frac{F_{A(j)}^{\left(z\right)}-F_{A(i)}^{\left(z\right)}}{1-e^{F_{A(i)}^{\left(z\right)}-F_{A(j)}^{\left(z\right)}}}$$

where A(i) denotes the amino acid encoded by codon *i*. At the root of the tree, for each site z, the sequence is drawn from the stationary distribution of the process specified by $\pi^{\left(z\right)}$, which is given by:

(15) $$\pi_{i}^{\left(z\right)}=Z^{\left(z\right)}\left[\prod_{k\in \{1,2,3\}}\sigma_{i[k]}\right]e^{F_{A(i)}^{\left(z\right)}},$$

where i[k] denotes the nucleotide at position k∈{1,2,3} of codon *i*, and $Z^{\left(z\right)}$ is the normalizing constant at site z:

(16) $$Z^{\left(z\right)}={\left(\sum_{j=1}^{61}\left[\prod_{k\in \{1,2,3\}}\sigma_{j[k]}\right]e^{F_{A(j)}^{\left(z\right)}}\right)}^{-1}$$

The substitution process is reversible and fulfills detailed balance conditions at each site z and between each pair of codons (*i*, *j*):

(17) $$\pi_{i}^{\left(z\right)}Q_{i,j}^{\left(z\right)}=\pi_{j}^{\left(z\right)}Q_{j,i}^{\left(z\right)}$$

Of note, by modeling fitness at the amino acid level, we assume that all codons encoding for one particular amino acid are selectively equivalent. In addition, in this modeling framework, the genetic code is of particular importance since the number of codons encoding for a particular amino acid varies greatly. As an example, tryptophan is encoded by one codon, whereas leucine is encoded by six codons. Intuitively, this variation makes the mutation bias more pronounced among codons encoding for the same amino acid, since there are more mutations possible that are selectively neutral (i.e., synonymous). On the other hand, the mutation bias is more constrained if the amino acid is encoded by few codons.

### Mean-Scaled Fixation Probability

The sequence at time *t* is denoted S(t) and the codon present at site z is denoted $S_{z}(t)$. For a given sequence, the mean-scaled fixation probability over mutations away from S(t), weighted by their probability of occurrence, is given by the ratio:

(18) $$\langle 2N_{e}P_{\text{fix}}(t)\rangle =\frac{\sum_{z=1}^{Z}\sum_{j\in N\left(S_{z}(t)\right)}Q_{S_{z}(t)\to j}}{\sum_{z=1}^{Z}\sum_{j\in N\left(S_{z}(t)\right)}\mu_{S_{z}(t)\to j}},$$

where N(i) is the set of nonsynonymous codons neighbors of codon *i* and $Q_{i,j}^{\left(z\right)}$ are defined as in equation (14). Averaged over all branches of the tree, the mean-scaled fixation probability is:

(19) $$\langle 2N_{e}P_{\text{fix}}\rangle =\int_{t}\langle 2N_{e}P_{\text{fix}}(t)\rangle dt,$$

where the integral is taken over all branches of the tree, whereas the integrand $\langle 2N_{e}P_{\text{fix}}(t)\rangle$ is a piece-wise function changing after every point substitution event. The mean-scaled fixation probability from weak (AT) to strong (GC) nucleotides, denoted $\langle 2N_{e}P_{\text{fix}}^{\text{WS}}\rangle$, is obtained similarly by restricting the sums (in the numerator and the denominator) from weak to strong mutations. A similar computation can be done from strong to weak.

### Derivation of MF Model

The MF codon model 〈Q〉 is defined such that $\langle Q_{i,j}\rangle$ is the average rate of substitution to codon *j*, conditional on currently being on codon *i*, the average being taken across sites. Importantly, sites differ in their probability of being currently in state *i*. The average should therefore be weighted by this probability.

Assuming an underlying site-specific mutation–selection process at equilibrium, given we know that a mutation is from codon *i*, the probability that this mutation is occurring at site z is:

(20) $$P(z|i)=\frac{\pi_{i}^{\left(z\right)}}{\sum_{z=1}^{Z}\pi_{i}^{\left(z\right)}}$$

The site-averaged (MF) substitution rate from codon *i* to *j* is as result given as:

(21) $$\left\langle Q_{i,j}\right\rangle =\sum_{z=1}^{Z}P(z|i)Q_{i,j}$$

If codon *i* and codon *j* are synonymous, this equation simplifies to the underlying mutation rate $R_{M(i,j)}$. Otherwise, if codon *i* and codon *j* are nonsynonymous, the MF substitution rate is:

(22) $$\langle Q_{i,j}\rangle =\langle R_{M(i,j)}2N_{e}P_{\text{fix}}(i,j)\rangle ,$$

(23) $$=R_{M(i,j)}\langle 2N_{e}P_{\text{fix}}(i,j)\rangle ,$$

(24) $$=R_{M(i,j)}\frac{\sum_{z=1}^{Z}\pi_{i}^{\left(z\right)}\frac{F_{A(j)}^{\left(z\right)}-F_{A(i)}^{\left(z\right)}}{1-e^{F_{A(i)}^{\left(z\right)}-F_{A(j)}^{\left(z\right)}}}}{\sum_{z=1}^{Z}\pi_{i}^{\left(z\right)}},$$

(25) $$=R_{M(i,j)}\frac{\sum_{z=1}^{Z}Z^{\left(z\right)}\frac{F_{A(j)}^{\left(z\right)}-F_{A(i)}^{\left(z\right)}}{e^{-F_{A(i)}^{\left(z\right)}}-e^{-F_{A(j)}^{\left(z\right)}}}}{\sum_{z=1}^{Z}Z^{\left(z\right)}e^{F_{A(i)}^{\left(z\right)}}}$$

As a result, $\langle 2N_{e}P_{\text{fix}}(i,j)\rangle$ is dependent on the source and target codon solely through the source amino acid (*x*) and target amino acid (*y*), hence the parameter $\omega_{x,y}$ identifies with the average fixation probability $\langle 2N_{e}P_{\text{fix}}\left(x\to y\right)\rangle$:

(26) $$\langle 2N_{e}P_{\text{fix}}\left(x\to y\right)\rangle =\frac{\sum_{z=1}^{Z}Z^{\left(z\right)}\frac{F_{y}^{\left(z\right)}-F_{x}^{\left(z\right)}}{e^{-F_{x}^{\left(z\right)}}-e^{-F_{y}^{\left(z\right)}}}}{\sum_{z=1}^{Z}Z^{\left(z\right)}e^{F_{x}^{\left(z\right)}}}.$$

### Mean-Scaled Fixation Probability $\langle 2N_{e}P_{\text{fix}}\rangle$ under the MF Model

The MF model is parameterized by a GTR mutation matrix R(σ,ρ) and the selection coefficient ω(β,ϵ). As a result, the mean-scaled fixation probability of nonsynonymous mutations is:

(27) $$\langle 2N_{e}P_{\text{fix}}\rangle =\frac{\sum_{i=1}^{61}\pi_{i}\sum_{j\in N\left(i\right)}^{\;}Q_{i,j}}{\sum_{i=1}^{61}\pi_{i}\sum_{j\in N\left(i\right)}^{\;}\mu_{i,j}},$$

(28) $$=\frac{\sum_{i=1}^{61}\left[\prod_{k\in \{1,2,3\}}^{\;}\sigma_{i[k]}\right]\epsilon_{A(i)}\sum_{j\in N\left(i\right)}R_{M(i,j)}\epsilon_{A(j)}\beta_{A(i),A(j)}}{\sum_{i=1}^{61}\left[\prod_{k\in \{1,2,3\}}\sigma_{i[k]}\right]\epsilon_{A(i)}\sum_{j\in N\left(i\right)}R_{M(i,j)}},$$

where i[k] denotes the nucleotide at position k∈{1,2,3} of codon *i*.

Similarly, the mean-scaled fixation probability from weak (AT) to strong (GC) nucleotides denoted $\langle 2N_{e}P_{\text{fix}}^{\text{WS}}\rangle$ is obtained similarly by restricting the sums (in the numerator and the denominator) to one nucleotide mutations only from weak to strong. Conversely, by restricting the sum from strong (GC) to weak (AT), we obtain $\langle 2N_{e}P_{\text{fix}}^{\text{SW}}\rangle$.

### Inference Method with Hyphy

Maximum likelihood estimation has been performed with the software Hyphy (Pond et al. 2005). The Python scripts generating the Hyphy batch files (for both MG and MF), as well as scripts necessary to replicate the experiments are available at https://github.com/ThibaultLatrille/NucleotideBias (last accessed January 31, 2021). 

## Supplementary Material

Supplementary data are available at *Molecular Biology and Evolution* online.

## Supplementary Material

msac005_Supplementary_Data
