# Supplementary material for: An Improved Codon Modeling Approach for Accurate Estimation of the Mutation Bias
Source: Mol Biol Evol. 2022 Jan 11;39(2):msac005. doi: 10.1093/molbev/msac005 (PMC8831783; doi:10.1093/molbev/msac005)
Supplement: msac005_Supplementary_Data [file msac005_supplementary_data.zip › figure3.pdf]

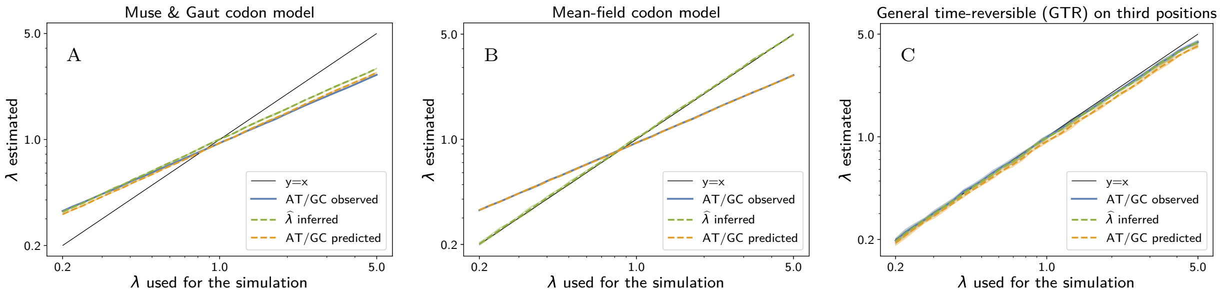

Muse & Gaut codon model
A
5.0
1.0
0.2
λ estimated
λ used for the simulation
y=x
AT/GC observed
$\hat{\lambda}$ inferred
AT/GC predicted
Mean-field codon model
B
General time-reversible (GTR) on third positions
C
