# Supplementary material for: An Improved Codon Modeling Approach for Accurate Estimation of the Mutation Bias
Source: Mol Biol Evol. 2022 Jan 11;39(2):msac005. doi: 10.1093/molbev/msac005 (PMC8831783; doi:10.1093/molbev/msac005)
Supplement: msac005_Supplementary_Data [file msac005_supplementary_data.zip › figure4.pdf]

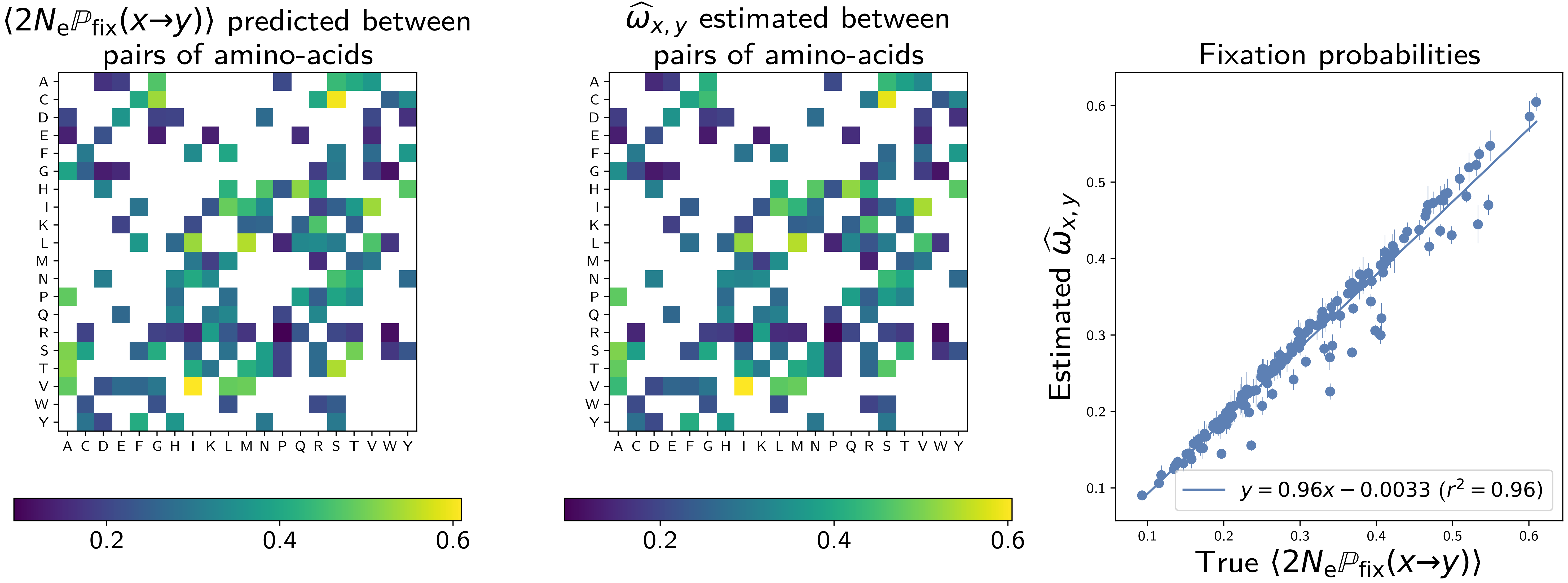

⟨2NeℙfixFix(x→y)⟩ predicted between pairs of amino-acids
ω̂x,y estimated between pairs of amino-acids
Fixation probabilities
A C D E F G H I K L M N P Q R S T V W Y
0.2 0.4 0.6
Estimated ω̂x,y
True ⟨2Neℙfix(x→y)⟩
y = 0.96x − 0.0033 (r2 = 0.96)
0.1 0.2 0.3 0.4 0.5 0.6
