# Supplementary material for: An Improved Codon Modeling Approach for Accurate Estimation of the Mutation Bias
Source: Mol Biol Evol. 2022 Jan 11;39(2):msac005. doi: 10.1093/molbev/msac005 (PMC8831783; doi:10.1093/molbev/msac005)
Supplement: msac005_Supplementary_Data [file msac005_supplementary_data.zip › supp-mat.pdf]

Supplementary materials

Contents

1 Simulated alignments **1**

1.1 Primate phylogeny - 4980 codons . . . 1

1.2 Primate phylogeny - 498 codons . . . 2

1.3 Primate phylogeny - 996 codons . . . 3

1.4 Primate phylogeny - 2490 codons . . . 4

1.5 Primate phylogeny - 9960 codons . . . 5

1.6 Primate phylogeny - 4980 codons - branch length %2 . . . 6

1.7 Primate phylogeny - 4980 codons - branch length x2 . . . 6

1.8 Primate phylogeny - 4980 codons - branch length x4 . . . 7

1.9 Primate phylogeny - 4980 codons - branch length x8 . . . 7

1.10 Mammalian phylogeny - 4980 codons . . . 8

1 Simulated alignments

1.1 Primate phylogeny - 4980 codons

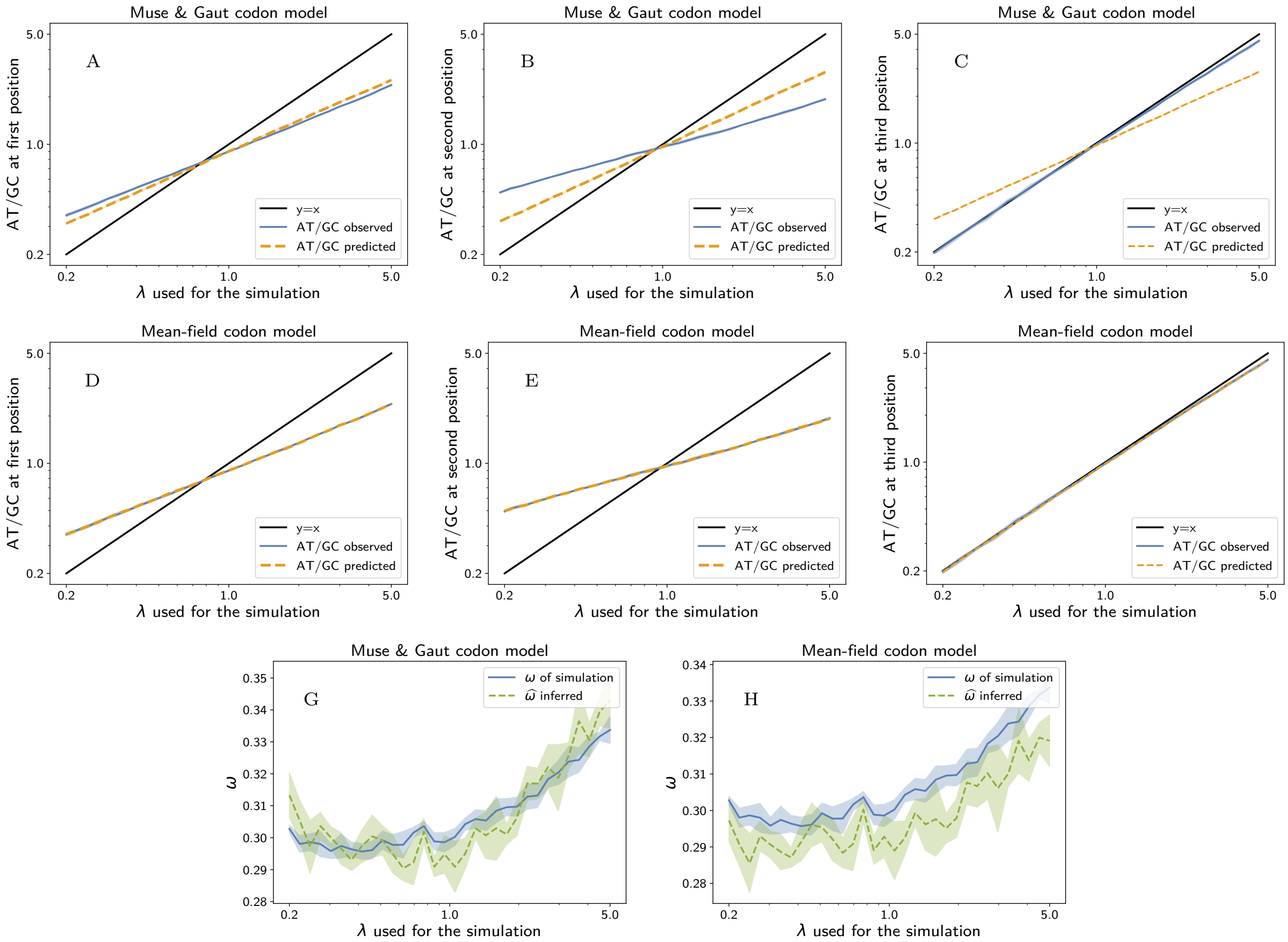

F

Simulations for 61 primate taxa and 9960 codon sites, for 32 different values of mutational bias (λ) from 0.2 to 5.0 with 5 replicates per value (simulations shown in the main manuscript). Observed AT/GC from the alignment and predicted AT/GC by the models at the first (panels A & D), second (panels B & E) and third (panels C & F) codon positions as a function of mutational bias for Muse & Gaut (panels A, B & C) and our tensor model

(panel C, D & E). Estimated $\hat{\omega}$ and simulated ω across replicates as a function of mutational bias (λ) for Muse & Gaut (panel G) and our tensor model (panel H).

1.2 Primate phylogeny - 498 codons

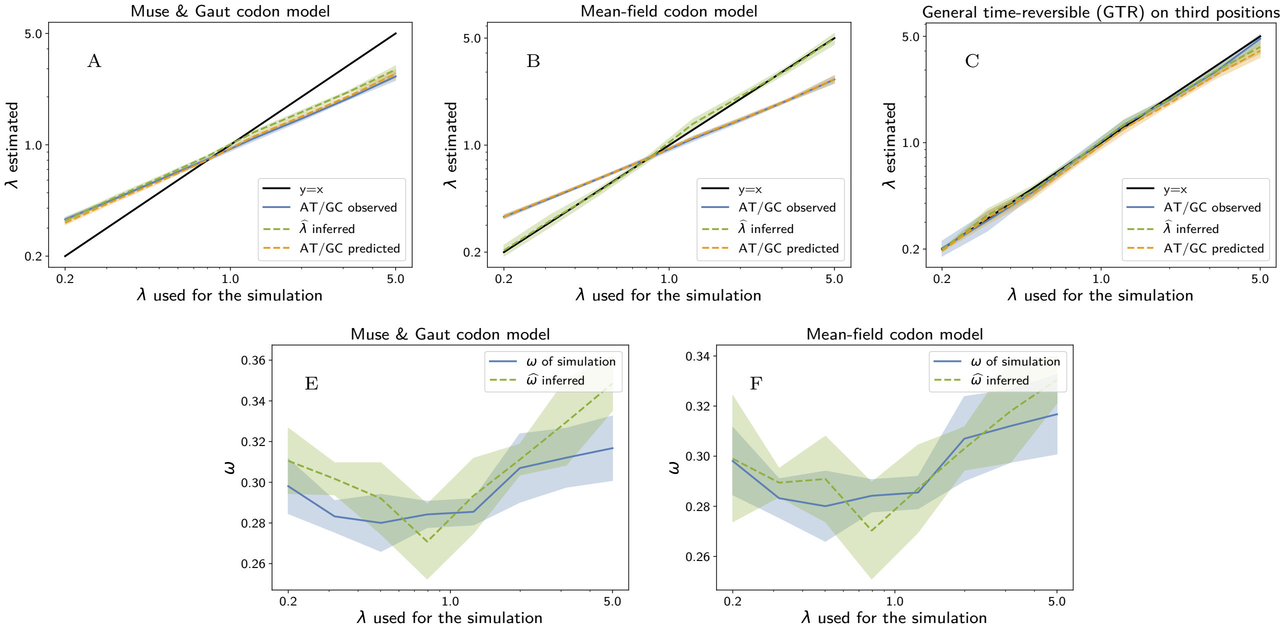

Simulations for 61 primate taxa and 498 codon sites, for 8 different values of mutational bias (λ) from 0.2 to 5.0 with 5 replicates per value. Estimated versus true mutational bias, using a codon model in which ω is modeled as a scalar (Muse & Gaut formalism, MG, panel A) or as a tensor (mean-field approach, panel B), or by applying a GTR nucleotide model to the 4-fold degenerate third-coding positions only (panel C). Estimated $\hat{\omega}$ and simulated ω across replicates as a function of mutational bias (λ) for Muse & Gaut (panel D) and our tensor model (panel E).

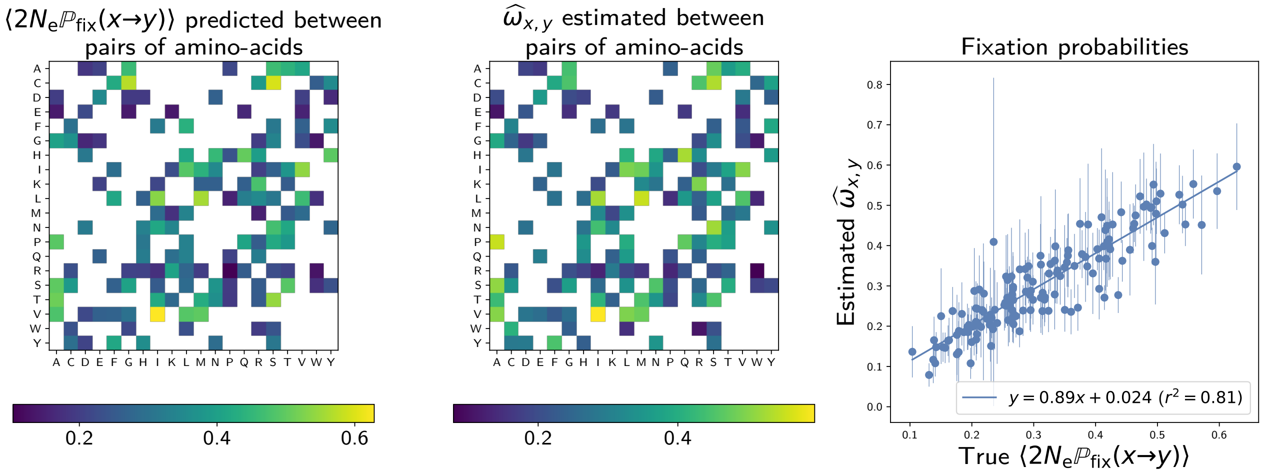

True versus estimated values of ω between pairs of amino-acids. Vertical bars are the 95% confidence intervals for the mean value.

1.3 Primate phylogeny - 996 codons

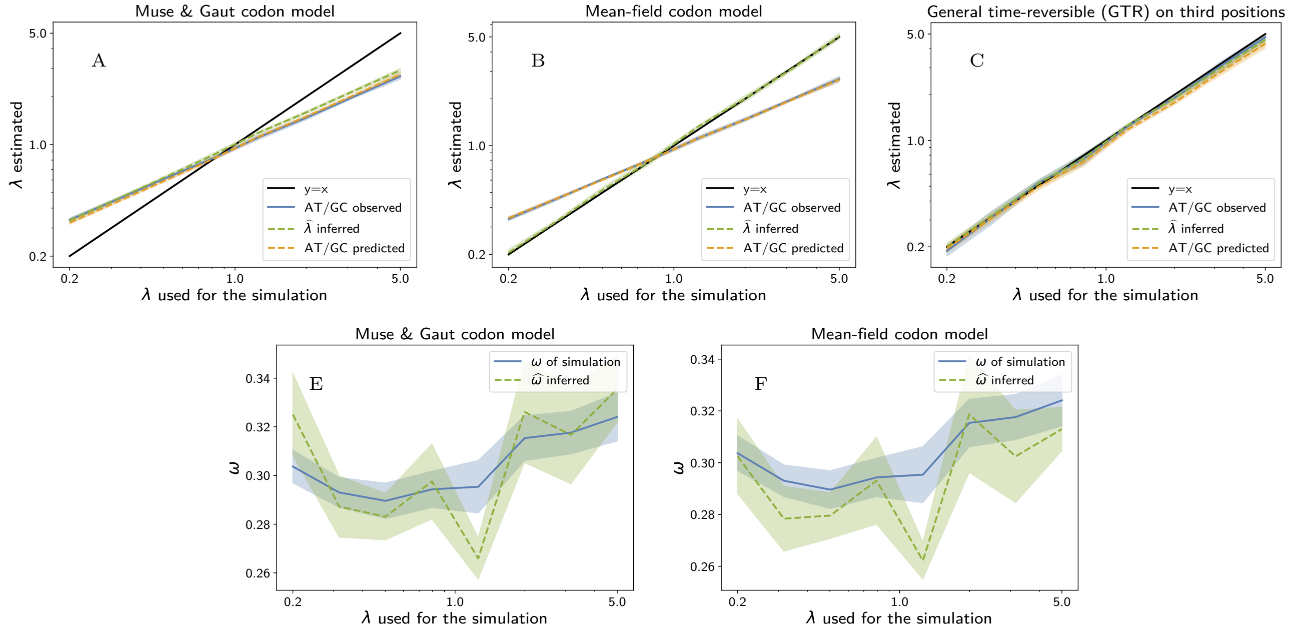

Simulations for 61 primate taxa and 996 codon sites, for 8 different values of mutational bias (λ) from 0.2 to 5.0 with 5 replicates per value. Estimated versus true mutational bias, using a codon model in which ω is modeled as a scalar (Muse & Gaut formalism, MG, panel A) or as a tensor (mean-field approach, panel B), or by applying a GTR nucleotide model to the 4-fold degenerate third-coding positions only (panel C). Estimated $\hat{\omega}$ and simulated ω across replicates as a function of mutational bias (λ) for Muse & Gaut (panel D) and our tensor model (panel E).

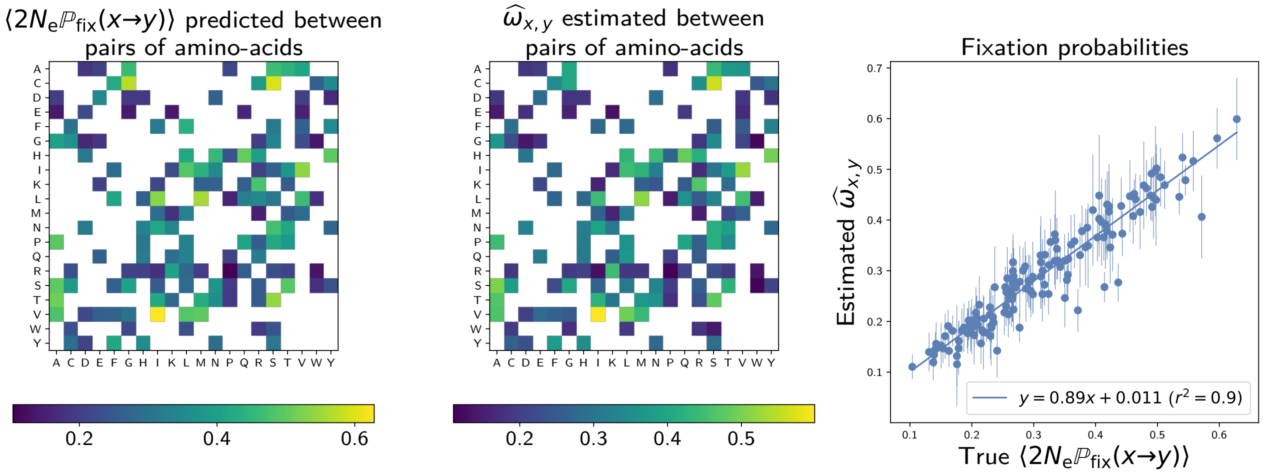

True versus estimated values of ω between pairs of amino-acids. Vertical bars are the 95% confidence intervals for the mean value.

1.4 Primate phylogeny - 2490 codons

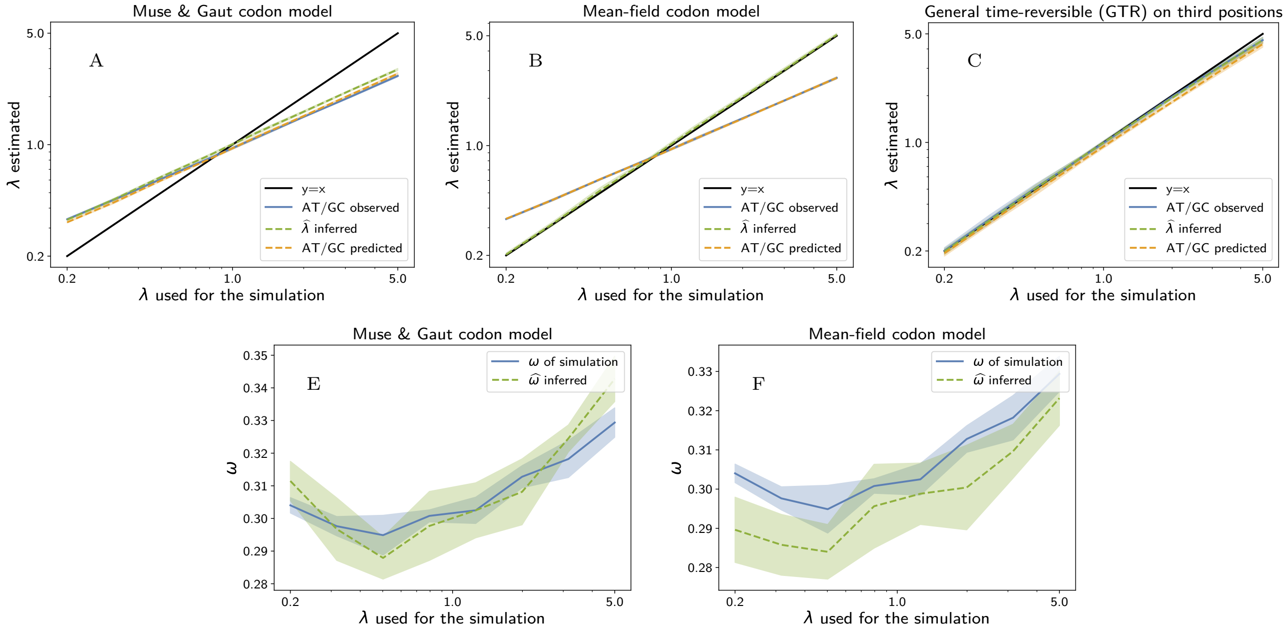

Simulations for 61 primate taxa and 2490 codon sites, for 8 different values of mutational bias (λ) from 0.2 to 5.0 with 5 replicates per value. Estimated versus true mutational bias, using a codon model in which ω is modeled as a scalar (Muse & Gaut formalism, MG, panel A) or as a tensor (mean-field approach, panel B), or by applying a GTR nucleotide model to the 4-fold degenerate third-coding positions only (panel C). Estimated $\hat{\omega}$ and simulated ω across replicates as a function of mutational bias (λ) for Muse & Gaut (panel D) and our tensor model (panel E).

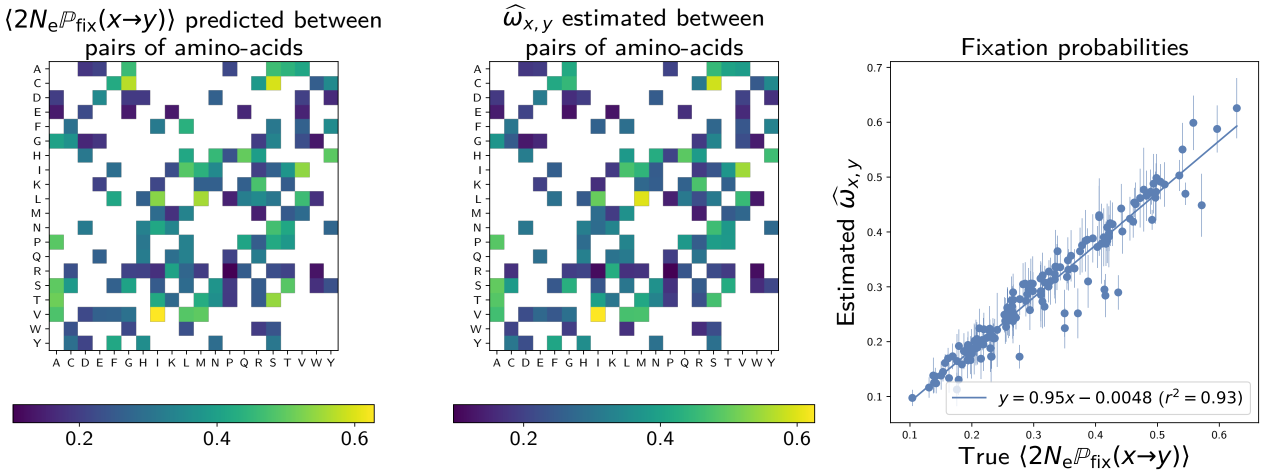

True versus estimated values of ω between pairs of amino-acids. Vertical bars are the 95% confidence intervals for the mean value.

1.5 Primate phylogeny - 9960 codons

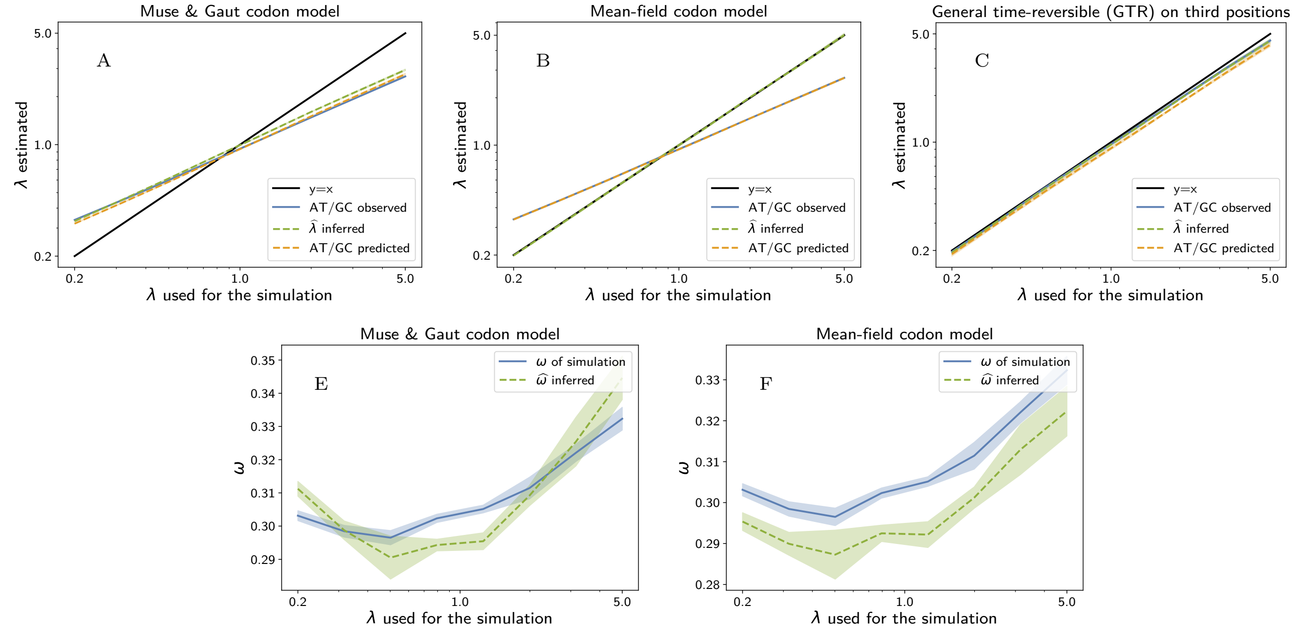

Simulations for 61 primate taxa and 9960 codon sites, for 8 different values of mutational bias (λ) from 0.2 to 5.0 with 5 replicates per value. Estimated versus true mutational bias, using a codon model in which ω is modeled as a scalar (Muse & Gaut formalism, MG, panel A) or as a tensor (mean-field approach, panel B), or by applying a GTR nucleotide model to the 4-fold degenerate third-coding positions only (panel C). Estimated $\hat{\omega}$ and simulated ω across replicates as a function of mutational bias (λ) for Muse & Gaut (panel D) and our tensor model (panel E).

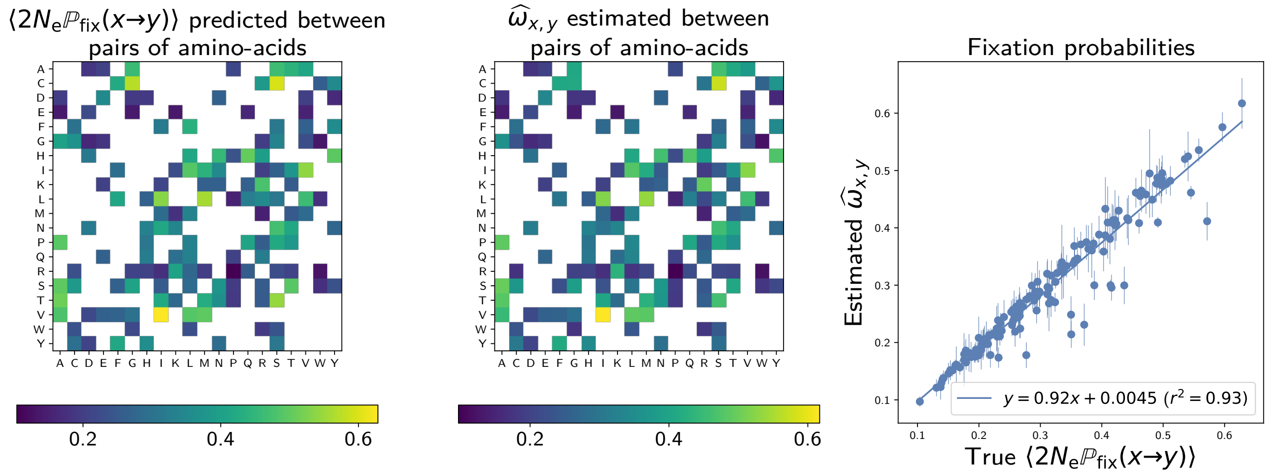

True versus estimated values of ω between pairs of amino-acids. Vertical bars are the 95% confidence intervals for the mean value.

1.6 Primate phylogeny - 4980 codons - branch length %2

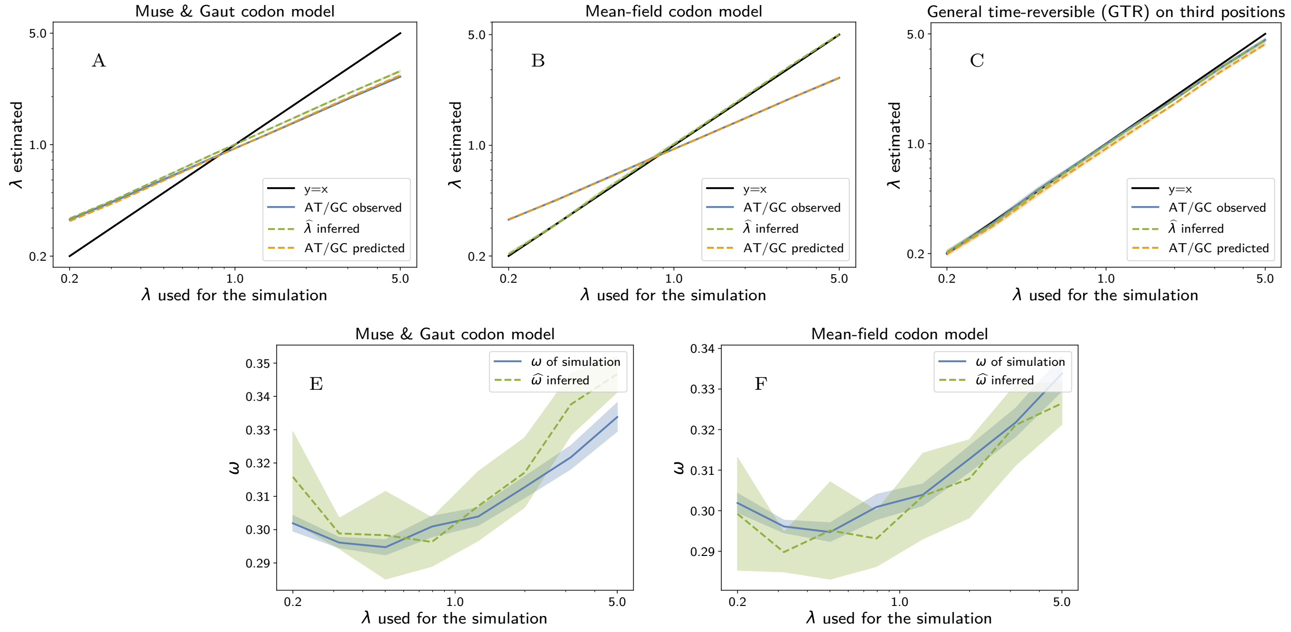

Simulations for 61 primate taxa with decreased branch lenght by a factor 2 and 4980 codon sites, for 8 different values of mutational bias (λ) from 0.2 to 5.0 with 5 replicates per value. Estimated versus true mutational bias, using a codon model in which ω is modeled as a scalar (Muse & Gaut formalism, MG, panel A) or as a tensor (mean-field approach, panel B), or by applying a GTR nucleotide model to the 4-fold degenerate third-coding positions only (panel C). Estimated $\hat{\omega}$ and simulated ω across replicates as a function of mutational bias (λ) for Muse & Gaut (panel D) and our tensor model (panel E).

1.7 Primate phylogeny - 4980 codons - branch length x2

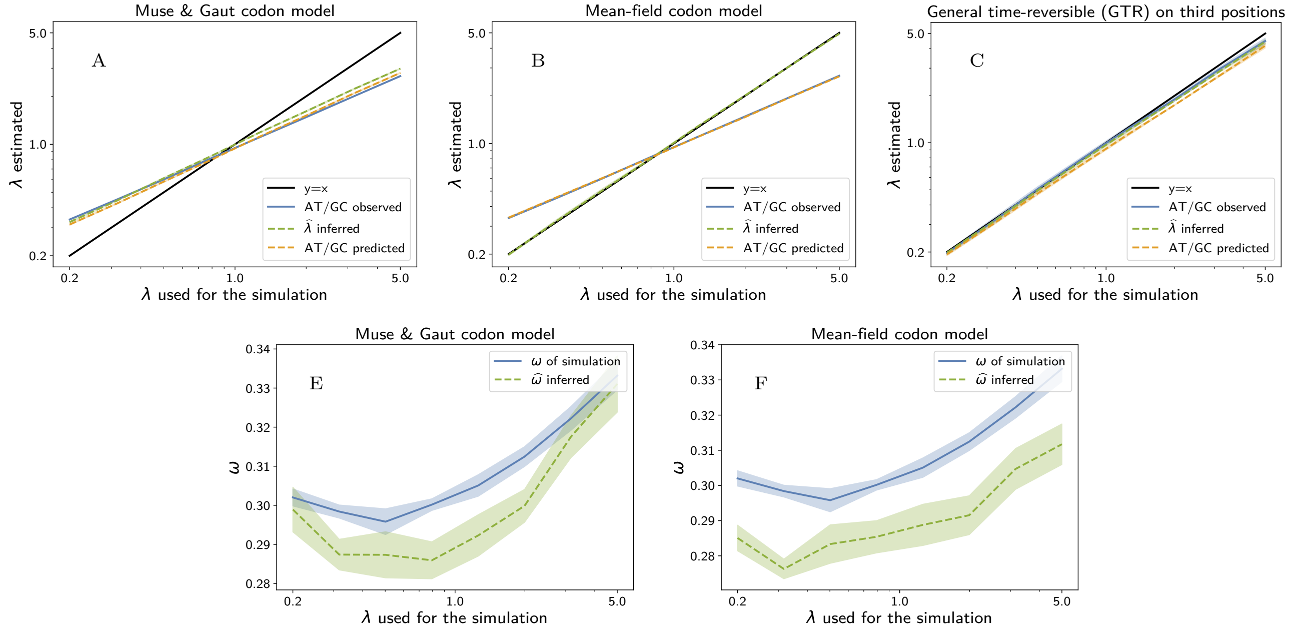

Simulations for 61 primate taxa with increased branch lenght by a factor 2 and 4980 codon sites, for 8 different values of mutational bias (λ) from 0.2 to 5.0 with 5 replicates per value. Estimated versus true mutational bias, using a codon model in which ω is modeled as a scalar (Muse & Gaut formalism, MG, panel A) or as a tensor (mean-field approach, panel B), or by applying a GTR nucleotide model to the 4-fold degenerate third-coding

positions only (panel C). Estimated $\hat{\omega}$ and simulated ω across replicates as a function of mutational bias (λ) for Muse & Gaut (panel D) and our tensor model (panel E).

1.8 Primate phylogeny - 4980 codons - branch length x4

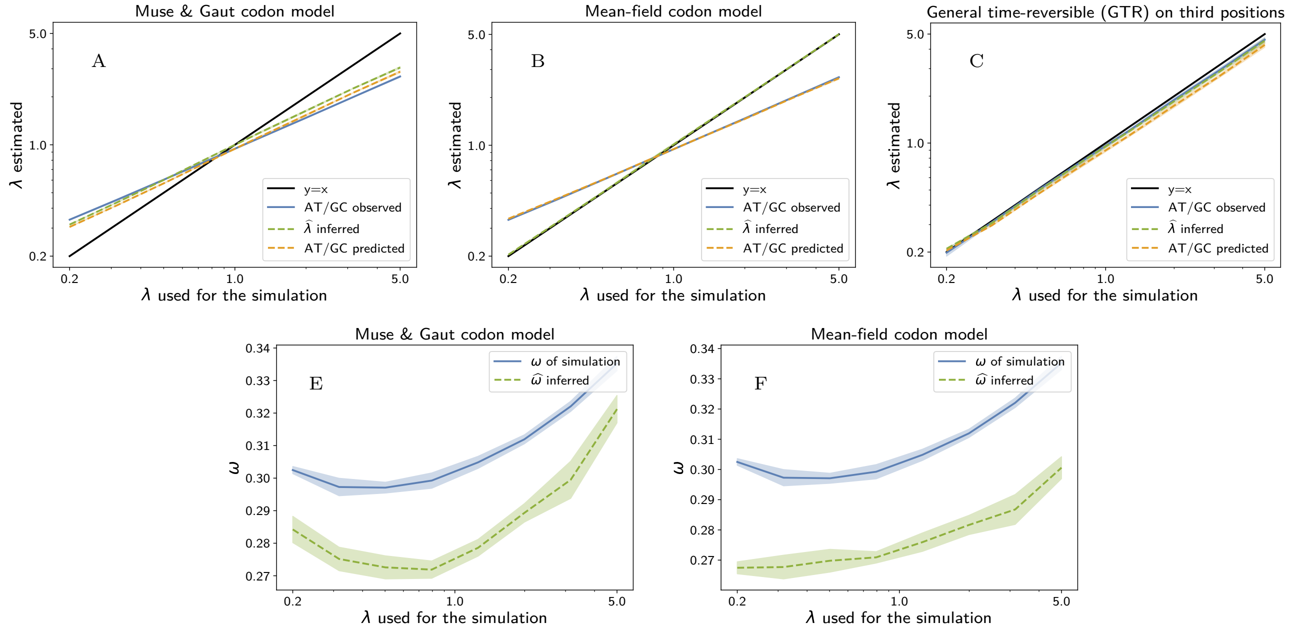

Simulations for 61 primate taxa with increased branch lenght by a factor 4 and 4980 codon sites, for 8 different values of mutational bias (λ) from 0.2 to 5.0 with 5 replicates per value. Estimated versus true mutational bias, using a codon model in which ω is modeled as a scalar (Muse & Gaut formalism, MG, panel A) or as a tensor (mean-field approach, panel B), or by applying a GTR nucleotide model to the 4-fold degenerate third-coding positions only (panel C). Estimated $\hat{\omega}$ and simulated ω across replicates as a function of mutational bias (λ) for Muse & Gaut (panel D) and our tensor model (panel E).

1.9 Primate phylogeny - 4980 codons - branch length x8

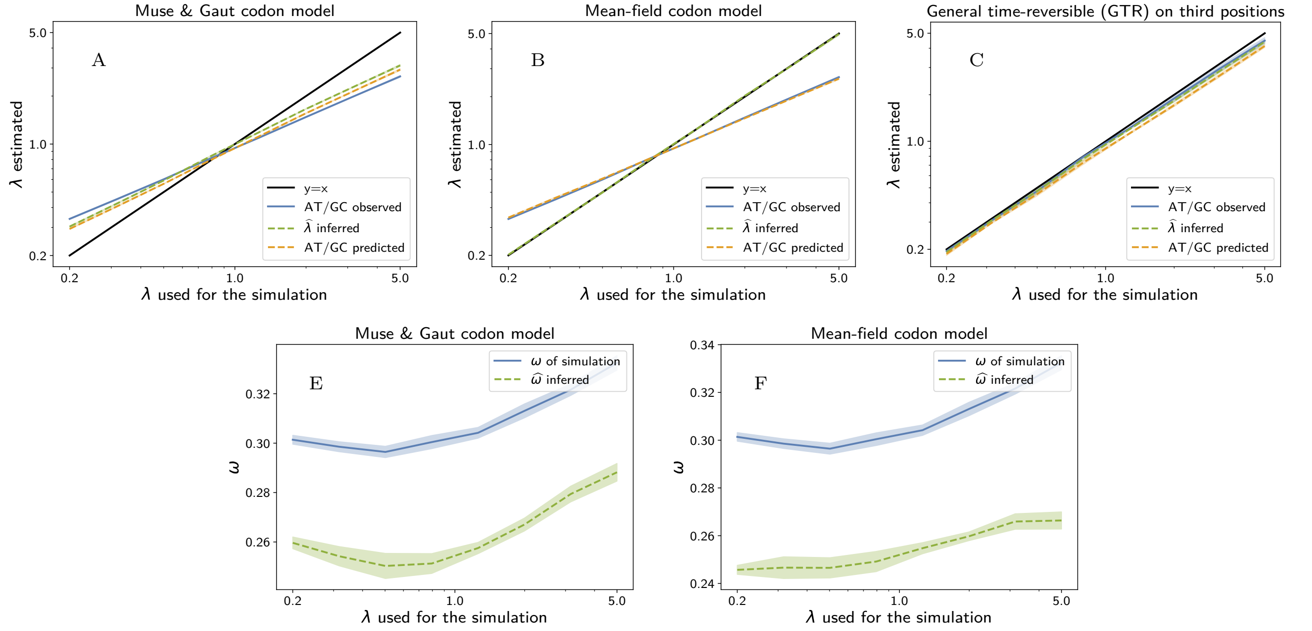

Simulations for 61 primate taxa with increased branch lenght by a factor 8 and 4980 codon sites, for 8 different values of mutational bias (λ) from 0.2 to 5.0 with 5 replicates per value. Estimated versus true mutational bias, using a codon model in which ω is modeled as a scalar (Muse & Gaut formalism, MG, panel A) or as a tensor (mean-field approach, panel B), or by applying a GTR nucleotide model to the 4-fold degenerate third-coding positions only (panel C). Estimated $\hat{\omega}$ and simulated ω across replicates as a function of mutational bias (λ) for Muse & Gaut (panel D) and our tensor model (panel E).

1.10 Mammalian phylogeny - 4980 codons

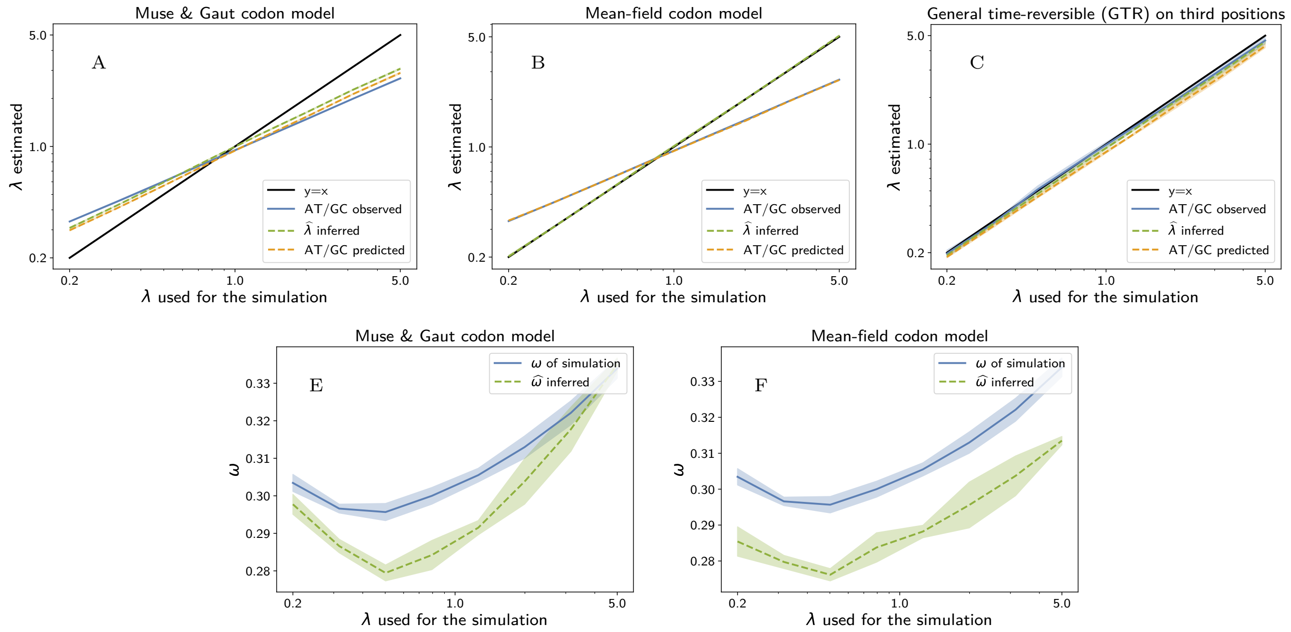

Simulations for 90 mammalian taxa and 4980 codon sites, for 8 different values of mutational bias (λ) from 0.2 to 5.0 with 5 replicates per value. Estimated versus true mutational bias, using a codon model in which ω is modeled as a scalar (Muse & Gaut formalism, MG, panel A) or as a tensor (mean-field approach, panel B), or by applying a GTR nucleotide model to the 4-fold degenerate third-coding positions only (panel C).
