# Supplementary figures and images for: An Improved Codon Modeling Approach for Accurate Estimation of the Mutation Bias
Source: Mol Biol Evol. 2022 Jan 11;39(2):msac005. doi: 10.1093/molbev/msac005 (PMC8831783; doi:10.1093/molbev/msac005)

Codon position 1

Codon position 2

Codon position 3

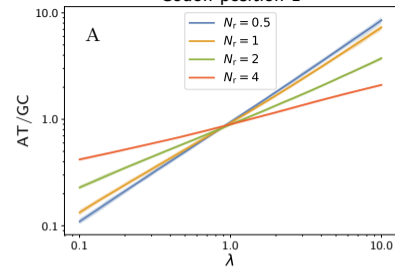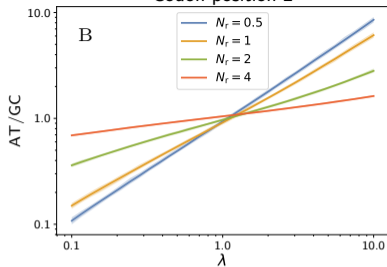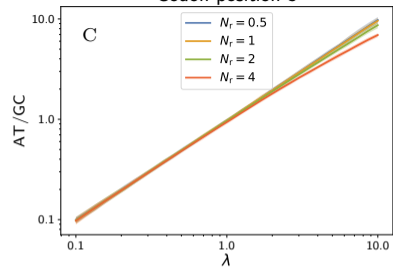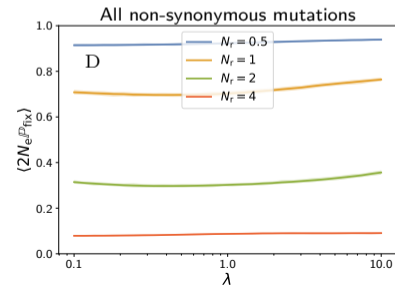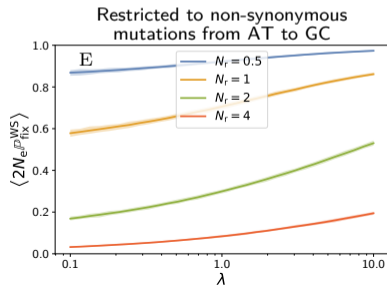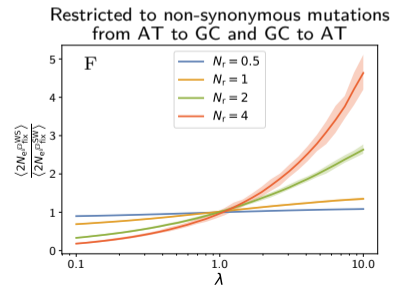

Supplement: msac005_Supplementary_Data [file msac005_supplementary_data.zip › figure1.pdf]

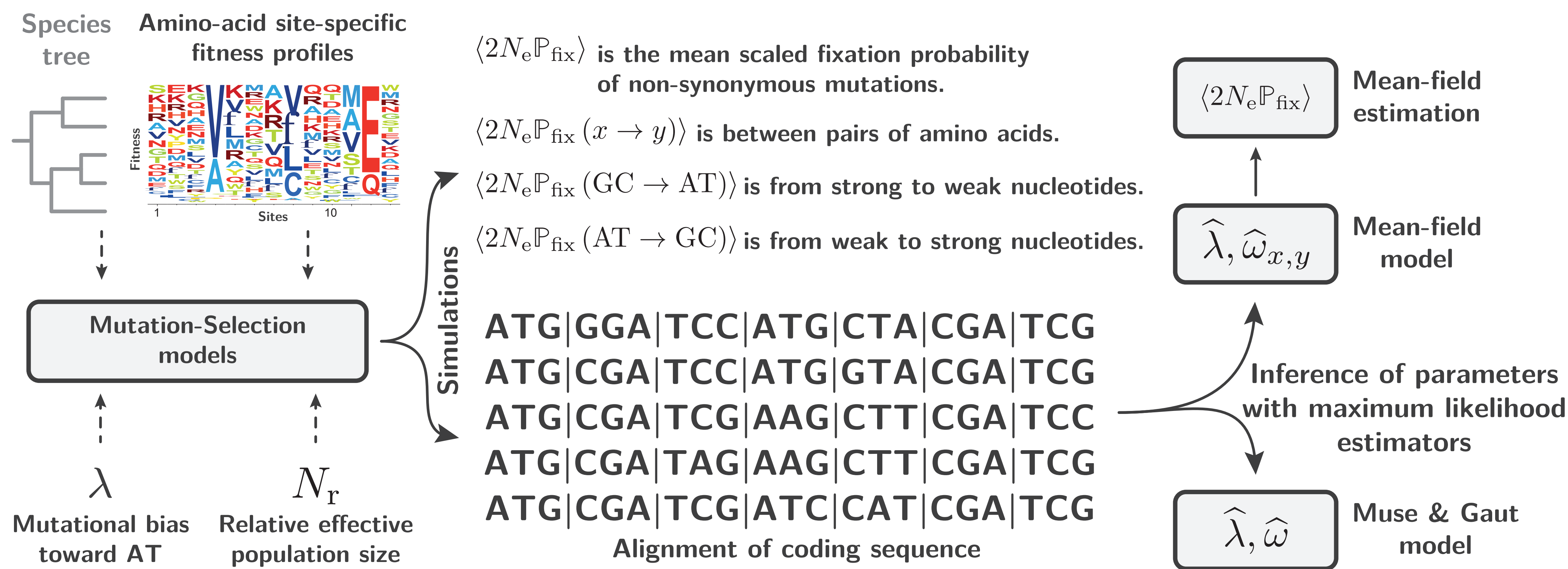

Supplement: msac005_Supplementary_Data [file msac005_supplementary_data.zip › figure2.pdf]
